# Does IR-loss promote plastome structural variation and sequence evolution?

**DOI:** 10.3389/fpls.2022.888049

**Published:** 2022-09-29

**Authors:** Zi-Xun Wang, Ding-Jie Wang, Ting-Shuang Yi

**Affiliations:** ^1^ Germplasm Bank of Wild Species, Kunming Institute of Botany, Chinese Academy of Sciences, Kunming, China; ^2^ Key Laboratory of Ministry of Education for Medicinal Plant Resource and Natural Pharmaceutical Chemistry, National Engineering Laboratory for Resource Developing of Endangered Chinese Crude Drugs in Northwest of China, College of Life Sciences, Shaanxi Normal University, Xi’an, China; ^3^ Kunming College of Life Science, University of Chinese Academy of Sciences, Kunming, China

**Keywords:** plastid genome evolution, structural variation, inverted repeat region loss, substitution rate, comparative genomics

## Abstract

Plastids are one of the main distinguishing characteristics of the plant cell. The plastid genome (plastome) of most autotrophic seed plants possesses a highly conserved quadripartite structure containing a large single-copy (LSC) and a small single-copy (SSC) region separated by two copies of the inverted repeat (termed as IR_A_ and IR_B_). The IRs have been inferred to stabilize the plastid genome *via* homologous recombination-induced repair mechanisms. IR loss has been documented in seven autotrophic flowering plant lineages and two autotrophic gymnosperm lineages, and the plastomes of these species (with a few exceptions) are rearranged to a great extent. However, some plastomes containing normal IRs also show high structural variation. Therefore, the role of IRs in maintaining plastome stability is still controversial. In this study, we first integrated and compared genome structure and sequence evolution of representative plastomes of all nine reported IR-lacking lineages and those of their closest relative(s) with canonical inverted repeats (CRCIRs for short) to explore the role of the IR in maintaining plastome structural stability and sequence evolution. We found the plastomes of most IR-lacking lineages have experienced significant structural rearrangement, gene loss and duplication, accumulation of novel small repeats, and acceleration of synonymous substitution compared with those of their CRCIRs. However, the IR-lacking plastomes show similar structural variation and sequence evolution rate, and even less rearrangement distance, dispersed repeat number, tandem repeat number, indels frequency and GC3 content than those of IR-present plastomes with variation in Geraniaceae. We argue that IR loss is not a driver of these changes but is instead itself a consequence of other processes that more broadly shape both structural and sequence-level plastome evolution.

## Introduction

Plastids, one of the major distinguishing characteristics of plant cells (Wicke et al., 2011), originated *via* endosymbiosis of a eukaryotic cell with a free-living cyanobacterial-like prokaryote (Margulis, 1988). The major role of plastids is performing photosynthesis, but they also have other functions including synthesis of starch, fatty acids, pigments, and amino acids (Neuhaus and Emes, 2000). Photosynthetic land plants usually have plastid genomes ranging from 120 kb to 160 kb in size and encoding ca. 80 protein-coding genes, 30 structural RNA genes, and four rRNAs (Wicke et al., 2011; Jansen and Ruhlman, 2012). The plastomes of most autotrophic plants have a highly conserved quadripartite structure containing two copies of large inverted repeat (IR) regions termed Inverted Repeat A (IR_A_) and Inverted Repeat B (IR_B_), and two single-copy (SC) regions termed the large single-copy (LSC) and small single-copy (SSC) regions (Wicke et al., 2011). The autotrophic plant IR typically contains a core set of four rRNA genes, six tRNA genes, and seven protein-coding genes (Bock, 2007; Wicke et al., 2011; Zhu et al., 2016).

Most autotrophic seed plants possess canonical IRs (Jin et al., 2020a); however, IR losses have been documented in seven autotrophic flowering plants lineages and two autotrophic gymnosperm lineages (Wu et al., 2011b; Lee et al., 2021). Plastomes of following lineages have lost their IR_A_: the Inverted Repeat-Lacking Clade (IRLC) of Leguminosae (Shinozaki et al., 1986); *Camoensia* of Leguminosae outside the IRLC (Lee et al., 2021); eight *Erodium* species of Geraniaceae (Guisinger et al., 2011; Blazier et al., 2016); *Carnegiea gigantea* (Sanderson et al., 2015) and *Lophocereus schottii* (unpublished data from GenBank) from Cactaceae; and the Cupressophyta composed of Cupressaceae, Taxaceae, Sciadopityaceae, Podocarpaceae and Araucariaceae (Wu and Chaw, 2014; Qu et al., 2017). Fewer lineages have experienced losses of the IR_B_ including the putranjivoid clade of Malpighiales (Jin et al., 2020a); two *Passiflora* species of Passifloraceae (Cauz-Santos et al., 2020); *Tahina spectabilis* of Arecaceae (Barrett et al., 2016); and Pinaceae (Wu et al., 2011a).

Plastomes of the IRLC and the putranjivoid clade are highly variable and rearranged to a great extent (Palmer and Thompson, 1982; Palmer et al., 1987; Jin et al., 2020a), supporting the idea that the IRs play an important role in stabilizing the structural integrity of the plastome *via* homologous recombination-induced repair mechanisms (Maréchal and Brisson, 2010; Guisinger et al., 2011). However, the plastomes of some species of the IRLC, such as alfalfa (*Medicago sativa *subsp.* sativa*) and *Wisteria floribunda*, show limited structural variation (Palmer et al., 1987; Lee et al., 2021). In addition, plastomes containing canonical IRs yet with significant plastome structural variation can be found in Campanulaceae (Cosner et al., 2004), Oleaceae (Lee et al., 2007), and *Pelargonium* (Geraniaceae; Chumley et al., 2006). Consequently, whether or not IRs play an important role in maintaining plastome stability remains controversial. This open issue could be addressed by comparing structural variation in taxa without the IR (IR-lacking), closely related taxa with plastome rearrangements that still have the IR (IR-present), and their closest relative(s) with canonical inverted repeats (CRCIRs). If IR-lacking plastomes have more structural changes than both IR-present and CRCIR plastomes, it would imply the loss of the IR is driving this structural instability. However, if both IR-lacking and IR-present plastomes have more structural changes than their CRCIRs, but do not differ significantly from each other in the number of structural rearrangements, it would imply the loss of the IR is not driving structural instability. Furthermore, previous studies have detected plastome-wide acceleration of substitution rates for IR-lacking species in Cupressoideae (Qu et al., 2017) and Fabaceae (Perry and Wolfe, 2002), but it is unclear if this acceleration has also occurred in other IR-lacking lineages.

Studies on the IR loss have typically focused on a specific lineage or just one or a few species, which prevents a broader understanding of the role of IRs in plastome structure and gene evolution. With the recent proliferation of sequenced plastomes, it is now possible to comprehensively study plastome evolution across all IR-lacking lineages of autotrophic seed plants to address fundamental questions about the function of the IR. We applied 84 plastomes (including 11 newly sequenced plastomes and 73 plastomes from GenBank) to compare structural variations (changes in gene and intron content, rearrangement distances, inversions and repeats) and sequence evolution (changes in substitution rates, selection pressure and GC content) between plastomes of all nine IR-lacking lineages and their closest relative(s) with canonical inverted repeats (CRCIRs) in a phylogenetic context.

## Materials and methods

### Representative sample selection

All nine lineages containing IR-lacking plastomes were sampled in this study. These include the angiosperm lineages IRLC (Leguminosae), *Camoensia* (Leguminosae), Geraniaceae, Cactaceae, Arecaceae, Passifloraceae, and the putranjivoid clade (including Putranjivaceae and Lophopyxidaceae), and the gymnosperm lineages of Cupressophyta (including Cupressaceae, Taxaceae, Sciadopityaceae, Podocarpaceae, and Araucariaceae; Stull et al., 2021) and Pinaceae. All species with IR-lacking plastomes of Arecaceae, Cactaceae, Geraniaceae, Passifloraceae, the putranjivoid clade, and *Camoensia* were included; up to eight species were sampled across each of the larger IR-lacking clades (i.e., the IRLC, the Cupressophyta, and Pinaceae) to represent major lineages. For each IR-lacking lineage, we sampled their closest relative(s) with canonical IRs for comparison. The canonical IRs means IRs have similar length and gene content as those of *Amborella trichopoda* (NC 005086).

### Sequence acquisition, assembly, and annotation

To obtain better representative IR-lacking and CRCIR plastomes, 11 new plastomes of *Anthyllis barba-jovis* (ON009079), *Coronilla valentina* subsp*. glauca* (ON009080), *Hebestigma cubense* var. *cubense* (ON009078), *Robinia pseudoacacia* (ON009076), *Afgekia filipes* (ON022041), *Astragalus bhotanensis* (ON009077), *Glycyrrhiza uralensis* (ON009073), *Hedysarum semenovii* (ON009074), *Maackia hupehensis* (ON009075) and *Camoensia* sp. (ON009081) from Leguminosae, and *Afrocarpus falcatus* (ON009072) of Podocarpaceae were determined based on the whole-genome Illumina sequencing dataset. We used the DNeasy Plant Mini Kit (Tiangen Biotech Co., Ltd., China) to extract total DNA and used NEBNext Ultra II DNA Library Prep Kit for Illumina (New England Biolabs, USA) to construct a short insert (350 bp) library. Paired-end (PE) sequencing with 2 × 150 bp was performed on Illumina HiSeq X TEN at Plant Germplasm and Genomics Center (Kunming Institute of Botany, China). Then the paired-end reads were filtered using Trimmomatic v.0.32 (Bolger et al., 2014) with default settings. Filtered reads were used to *de novo* assembly plastomes using GetOrganelle v.1.6.2a with default settings (Jin et al., 2020b). The final assembly results were checked in Bandage (Wick et al., 2015). We also downloaded 73 additional plastomes from GenBank (https://www.ncbi.nlm.nih.gov; **Table S1**; GenBank accessed August 30th, 2021). We initially used PGA (Qu et al., 2019) and GeSeq (https://chlorobox.mpimpgolm.mpg.de/geseq.html; Tillich et al., 2017) to annotate all plastomes with *Amborella trichopoda* (NC_005086) as the reference (Goremykin et al., 2003). Finally, the annotations generated by PGA were manually adjusted in Geneious Prime v.2020.0.5 (Kearse et al., 2012).

### Phylogenetic analyses

Phylogenetic analyses of the nine lineages, all IR-lacking species and their CRCIRs, 21 IR-lacking species listed in **Table 1** that have dispersed repeats at boundary regions of inversions, 22 Geraniaceae species listed in **Table S5** were performed using plastome protein-coding and rRNA genes that were present in all examined species. We used get_annotated_regions_from_gb.py (https://github.com/Kinggerm/PersonalUtilities; Zhang et al., 2020) to extract and separate gene sequences (protein-coding genes, and rRNA genes) and intergenic regions, and used MAFFT v. 7.305b (Katoh et al., 2005) to conduct alignment of each region, then used concatenate_fasta.py (https://github.com/Kinggerm/PersonalUtilities; Zhang et al., 2020) to concatenate sequences into a data set. We reconstructed phylogenetic trees and assessed the branch support using RAxML version 8.1.21 with 1000 bootstrap replicates and the “GTRGAMMA” model (Stamatakis, 2014); results were visualized in FigTree v.1.4.4.

**Table 1:** Statistics of dispersed repeats and their corresponding inversions.

Species	Inversion Site	Inversion Length	Repeat Length
*Erodium carvifolium*	30710–36608	5898	445
*Erodium reichardii*	5647–30221	24574	352
*Erodium texanum*	23425–46586	23161	3359
*Drypetes longifolia*	5605–89001	83396	1260
*Drypetes hainanensis*	5474–89321	83847	1191
*Drypetes indica*	5529–90486	84957	1047
*Drypetes lateriflora*	6031–90468	84437	1484
*Drypetes similis*	5716–89014	83298	1221
*Drypetes diopa*	5967–89759	83792	1357
*Drypetes chevalieri*	5917–90107	84190	1398
*Passiflora capsularis*	80769–112976	32207	791
*Passiflora costaricensis*	77218–114228	37010	1070
	77218–84722	7504	280
*Taiwania cryptomerioides*	7164–41842	34678	271
*Glyptostrobus pensilis*	46836–123831	76995	274
*Sciadopitys verticillata*	7855–47024	39169	157
*Prumnopitys andina*	32987–110212	77225	152
*Picea abies*	8507–32712	24205	395
*Abies fargesii*	7812–53059	45247	1176
*Larix gmelinii*	9126–51283	42157	411,44
*Keteleeria davidiana*	8159–50365	42206	610,66,15
*Pinus armandii *	49465–50435	970	232

### Repeat analyses

We used the Find Repeats plugin of Geneious Prime to detect dispersed repeats; the minimum repeat length was set as 30 bp and the maximum mismatch was set as 0% (Jin et al., 2020a). To ensure the accuracy of the detection results, we excluded repeats up to 10 bp longer than the contained repeat and excluded contained repeats when longer repeat has the frequency of at least three.

We also used MISA (https://webblast.ipk-gatersleben.de/misa; Thiel et al., 2003) with preset parameters followed by the core PERL function MISA.pl to identify the tandem repeats. The repeat units are one (mononucleotide), two (di-) nucleotides, three (tri-) nucleotides, four (tetra-) nucleotides, five (penta-) nucleotides, and hexanucleotides. The parameters were set as follows:

Definition (unit size, min repeats): 1-10 2-6 3-5 4-5 5-5 6-5, Interruptions (max_difference_between_2_SSRs): 100 (default setting). For the CRCIRs with the normal quadripartite structure, to avoid redundancy for the repeat identification, one IR copy was excluded for this analysis.

### Comparative analyses of genome structure

We used the progressiveMauve algorithm in Mauve v2.3.1 (Darling et al., 2010) with default settings to build whole plastome alignments for each of the nine lineages and its CRCIRs, respectively. For optimal homology assessment, one IR copy was excluded from plastomes with both IRs (Wicke et al., 2013). The strand orientation of each Locally Collinear Block (LCBs) was determined compared with those of their CRCIRs, and each LCB is then assigned a number and direction (+/-; Jin et al., 2020a). Subsequently, we used GRIMM (http://grimm.ucsd.edu/cgi-bin/grimm.cgi; Tesler, 2002) to calculate plastome rearrangement distances, the minimum number of inversions were required to transform the LCB orders from plastome of reference species (with yellow background in **Table S1**) to the target plastomes of IR-lacking species and their CRCIRs. The orientation of the SSC is arbitrary in IR-present plastomes (Walker et al., 2015), which is different in the IR-lacking plastomes and plastomes of their CRCIRs of two Leguminosae groups, we then manually reversed SSC in plastomes of CRCIRs of two Leguminosae groups to eliminate the arbitrary affection.

### Molecular evolutionary analyses

The synonymous substitution rates of protein-coding genes of whole plastomes, single-copy regions, and IR regions were analyzed using HyPhy v.2.2 (Pond et al., 2005) with the MG94×GTR_3 × 4 codon model, respectively. The protein-coding genes of the single-copy (SC) regions were classified into 11 data sets according to their functions including *atp* genes, *chl* genes, *ndh* genes, *pet* genes, *psa* genes, *psb* genes, *rpl* genes, *rpo* genes, *rps* genes, other housekeeping genes (other-HK: *accD*, *clpP*, *matK*, *ycf1*, *ycf2*, and *ycf12*) and other photosynthesis genes (other-PS: *ccsA*, *cemA*, *rbcL*, *ycf3*, and *ycf4*). We also extracted IR region protein-coding genes (*rps12*, *rps7*, *ndhB*, *ycf2*, *ycf1*, *rpl23*, *rpl2* of angiosperm species; *ycf2*, *ndhB*, *rps12*, *rps7* of gymnosperms species). These protein-coding gene alignments were generated using MAFFT v.7.305b (Katoh et al., 2005), and were used to constrain nucleotide alignments using PAL2NAL (Suyama et al., 2006).

We assessed the direction and the strength of changes of selection pressure across functional genes (using the same classification as above for synonymous substitution rates) *via* the nonsynonymous/synonymous rate ratio (also called “omega” and denoted by ω or *d_N_*/*d_S_*) calculated using branch models in a phylogenetic framework (**Figures S1A-I**). We tested different hypotheses using branch models in EasyCodeML v.1.21 (Gao et al., 2019) that allow ω to vary among branches in the tree. Omega in selected branches (foreground) was then compared with omega in unselected branches (background) in each of the nine lineages, respectively. When ω < 1, the sequence is under purifying selection, and when ω > 1, the sequence is under positive selection. If selection pressure is relaxed, ω will move toward 1, which means, under purifying selection, the smaller the value, the more relaxed; and under positive selection, the larger the value, the more relaxed (Wicke et al., 2016). For each terminal branch of the phylogeny, ω was calculated using the free-ratios branch model (model = 1) implemented in the codeml program from the PAML package v.4.8a (Goldman and Yang, 1994; Yang, 2007; De La Torre et al., 2017).

### Microstructural changes and single nucleotide variation

We classified sequences into three data sets (protein-coding genes, rRNAs, and intergenic regions), then used DnaSP v.6 (Rozas et al., 2017) to identify the frequency (number of mutated bases/number of total plastome bases) of short insertions or deletions (indels) involved in microstructural changes (Yamane et al., 2006; Wicke et al., 2016). We used SNP-sites v. 2.5.1 (Page et al., 2016) to count the frequency of single nucleotide variation sites (SNVs). Both analyses compared each plastome of the IR-lacking species and their CRCIRs with the plastome of the reference species (with yellow background in **Table S1**), respectively.

### GC content and codon usage

We employed the program CodonW v.1.4.4 (http://codonw.sourceforge.net/; Guisinger et al., 2011) to conduct the analyses of relative synonymous codon usage (RSCU), total guanine-cytosine (GC) content, and guanine-cytosine content at third codon position (GC3). RSCU values indicate codon usage bias in protein-coding sequences; RSCU values >1.00 indicates that the codons used more frequently than expected, while RSCU values < 1.00 indicates codons used less frequently than expected.

### Statistical analysis

The ape and nlme R package (Paradis and Schliep, 2019; Pinheiro et al., 2022) were used to perform the phylogenetic t-test and phylogenetic least squares regression (PGLS) to determine whether any of these metrics differed between the IR-lacking plastomes and those of their CRCIRs while taking phylogenetic relatedness into account. Based on the phylogenetic tree of all IR-lacking species and their CRCIRs (**Figure S1J**), the phylogenetic t-test to account for variances of dispersed repeat number, tandem repeat number, rearrangement distance, indels frequency, SNVs frequency, whole plastome GC content, protein-coding genes GC content, GC3 content, synonymous substitution rate between IR-lacking plastomes and those of CRCIRs was performed by coding the categorical variable as a dummy quantitative variable with value 0 for IR-lacking species and 1 for CRCIRs following Organ et al. (2007) and Charboneau et al. (2021). Following Organ et al. (2007), PGLS was used to determine whether there is a correlation between two continuous variables of dispersed repeat length and inversion length based on the phylogenetic tree of 21 species listed in **Table 1** (**Figure S1K**), dispersed number and rearrangement distance, as well as ω and rearrangement distance based on the phylogenetic tree of all IR-lacking species and those of their CRCIRs (**Figure S1J**).

To test if IR can drive plastome structural variation, the phytools R package (Revell, 2012) was used to perform a phylogenetic ANOVA with *post-hoc* tests based on the phylogenetic tree of 22 species listed in **Table S5** (**Figure S1L**) on rearrangement distance, repeat number, indels frequency, SNVs frequency, whole plastome GC content, GC3 content and synonymous substitution rates between eight IR-lacking plastomes from *Erodium* and all six structural variable IR-present plastomes of *Geranium* in Geraniaceae taking phylogenetic relatedness into account.

## Results

### Plastome features in nine lineages

All 11 newly sequenced plastomes were submitted to the GenBank (ON009072–ON009081 and ON022041; **Table S6**). Specific characteristics of plastomes from the nine lineages are described below. Relative to those of their CRCIRs (with the plastome size of 124,858 bp–139,924 bp after removing one IR), the plastome length of the IR-lacking species was usually shorter, ranging from 113,064 bp in *Carnegiea gigantea* of Cactaceae to 145,625 bp in *Agathis dammara* of the Cupressophyta (**Table S1**). Consistent with previous studies, plastomes of the Cupressophyta, two Leguminosae lineages, two species of Cactaceae, and eight *Erodium* species had lost their IR_A_, while those of Pinaceae, two species of Passifloraceae, the putranjivoid clade, and *Tahina spectabilis* of Arecaceae had lost IR_B_ (**Figure S1J**).

Gene content was variable in plastomes of the IR-lacking species (details shown in **Table S1**). *Tahina spectabilis* (Arecaceae) had the largest number of protein-coding genes (i.e., 79, the same number as its CRCIRs). In contrast, the other IR-lacking lineages had different degrees of gene loss or pseudogenization, typically involving *accD*, *infA*, *rpl20*, *rpl22*, *rpl23*, *rpl33*, *rps7*, *rps12*, *rps16*, *ycf1*, *ycf2* and/or *ycf12*. Among these genes, the most frequently lost gene was *rps16* and *rpl20*, *rpl22*, *rpl23* and *rpl33*. For IR-lacking plastomes of Cactaceae and Pinaceae, multiple *ndh* genes (*ndhA*~*ndhK*) were lost and/or became pseudogenes (**Table S1**). Except for *T. spectabilis*, other species lost the *clpP* intron 1 or both intron 1 and 2 (details shown in **Table S1**); the *rps12* intron at the 3’ portion was lost in IRLC, the putranjivoid clade, and *Passiflora*; the *atpF* intron was lost in the putranjivoid clade and *Passiflora* (**Table S1**). Many gene duplication events also occurred in the IR-lacking species (**Table S1**), including eight tRNA genes (*trnD-GUC*, *trnG-GCC*, *trnH-GUG*, *trnI-CAU*, *trnQ-UUG*, *trnS-GCU*, *trnT-GGU* and *trnV-GAC*), and five protein-coding genes (*psbK*, *psbI*, *psaM*, *rpl33* and *ycf12*).

### Length and quantity of repeats

**Figure 1:**
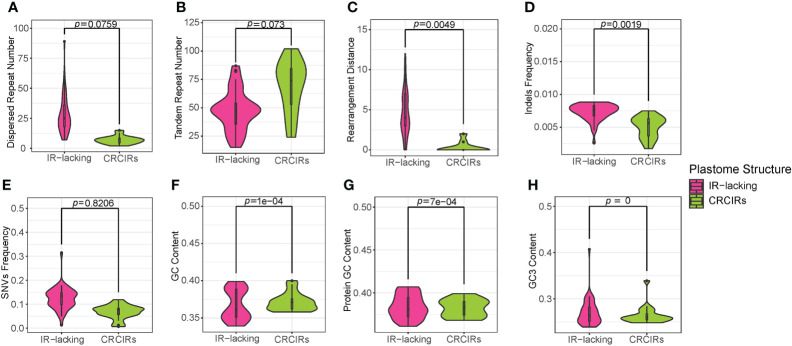
Violin plots for comparisons of eight variables between IR-lacking plastomes and those of their CRCIRs with phylogenetic t-test. **(A)** dispersed repeat number; **(B)** tandem repeat number; **(C)** rearrangement distance; **(D)** indels frequency; **(E)** SNVs frequency; **(F)** whole plastome GC content; **(G)** protein-coding genes GC content; **(H)** GC3 content.

We observed that most plastomes of IR-lacking species had more dispersed repeats and larger dispersed repeats than those of their CRCIRs (**Figures 1A**, **S6**). *Tahina spectabilis* had 3 more dispersed repeats than the average of its CRCIRs; Cactaceae averaged 27 more dispersed repeats than their CRCIRs; Geraniaceae averaged 12.6 more dispersed repeats than their CRCIRs; IRLC averaged 15.3 more dispersed repeats than their CRCIRs; the putranjivoid clade averaged 17.8 more dispersed repeats than their CRCIRs; Passifloraceae averaged 35 more dispersed repeats than their CRCIRs; the Cupressophyta averaged 41.1 more dispersed repeats than their CRCIRs; Pinaceae averaged 24.9 more dispersed repeats than their CRCIRs; *Camoensia* averaged 4.5 fewer dispersed repeats than its CRCIRs (**Table S2**). Plastomes of all *Drypetes* species, one *Passiflora* species (*Passiflora costaricensis*), two *Erodium* species (*Erodium trifolium* and *Erodium texanum*), and three Pinaceae species (*Abies fargesii*, *Cedrus deodara*, and *Pseudolarix amabilis*) possessed a pair of dispersed repeats with a length of more than 1000 bp (**Table S2**).

Contrary to these increases in dispersed repeats, plastomes of most IR-lacking species showed decreases in tandem repeats compared to those of their CRCIRs (**Figures 1B**, **S6**). *Tahina spectabilis* had 11.3 fewer tandem repeats than the average of its CRCIRs; Cactaceae averaged 37 fewer tandem repeats than their CRCIRs; Geraniaceae averaged 19.5 fewer tandem repeats than their CRCIRs; IRLC averaged 23.4 fewer tandem repeats than their CRCIRs; *Camoensia* averaged 27 fewer tandem repeats than its CRCIRs; the putranjivoid clade averaged 44 fewer tandem repeats than their CRCIRs; the Cupressophyta averaged 0.125 fewer tandem repeats than their CRCIRs; Pinaceae averaged 17.6 fewer tandem repeats than their CRCIRs; Passifloraceae averaged 3.5 more tandem repeats than their CRCIRs (**Table S2**).

### Plastome major structural variation

The plastome structure of most IR-lacking species underwent greater rearrangement compared with that of their CRCIRs (**Figures 1C**, **S2**, **S6**). The rearrangement distance of IR-lacking plastomes was 0–12, compared with 0–2 in their CRCIRs (**Table S2**). The plastome of *E. texanum* plastome had the largest rearrangement distance and three IRLC species (*Afgekia filipes*, *Astragalus bhotanensis*, *Cicer arietinum* and *Glycyrrhiza uralensis*) had the smallest in all IR-lacking species. Subsequently, we found one to three pairs of dispersed repeats at boundary regions (within 500 bp from the endpoints) of 21 of the 146 inversions (**Table 1**), and the length of such dispersed repeats had a significant positive correlation with the length of inversions (**Figure 3A**). We also observed an interesting phenomenon in IR-lacking plastomes: no matter how complex their plastomes were in terms of structural variation, the original six genes in the IR (*rrn16*, *trnI-GAU*, *trnA-UGC*, *rrn23*, *rrn4.5*, and *rrn5*) always maintained the same order and direction.

### Nucleotide substitution rates

**Figure 2:**
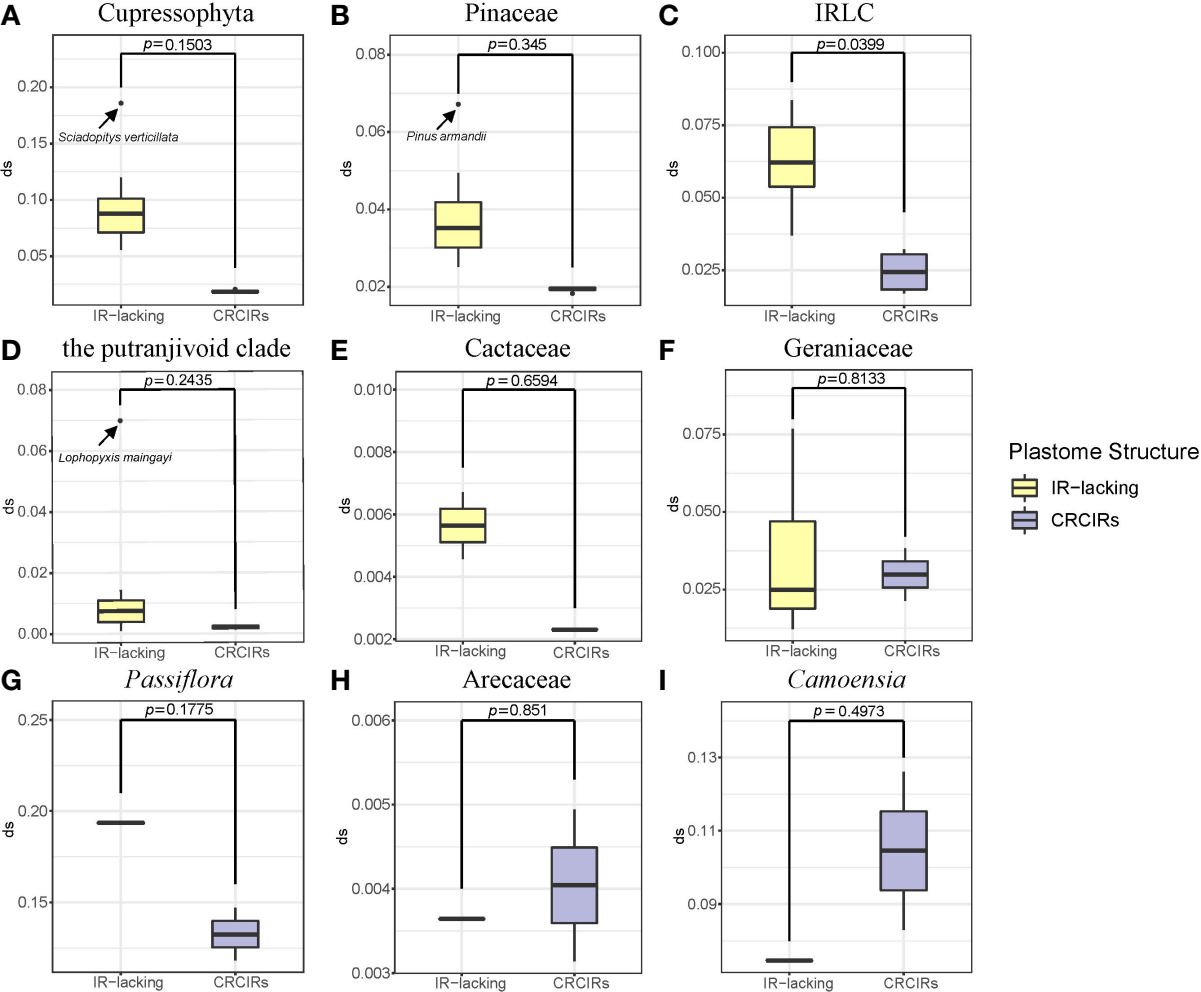
Boxplots for comparisons of synonymous substitution rates of whole protein-coding genes between IR-lacking plastomes and those of their CRCIRs with phylogenetic t-test. **(A)** Cupressophyta; **(B)** Pinaceae; **(C)** IRLC; **(D)** the putranjivoid clade; **(E)** Cactaceae; **(F)** Geraniaceae; **(G)** *Passiflora*; **(H)** Arecaceae; **(I)** *Camoensia*. Thick lines within boxes are medians, and outliers are shown as circles.

Except for *T. spectabilis*, *C. scandens*, and six *Erodium* species, the synonymous substitution rates of protein-coding genes in most IR-lacking species were higher compared with those of their CRCIRs (**Figures 2**, **S6**), which are 1.1 times to 6.9 times greater (*d*_S_IR-lacking/*d*_S_IR-normal) than those of their CRCIRs (**Table S2**). Eleven different kinds of protein-coding genes in the single-copy (SC) region of the nine lineages showed different degrees of higher or lower synonymous substitution rates ranging from 5 times less to 12 times greater than those of their CRCIRs (show detail in **Figure S3** and **Table S2**). Except for the *ndhB* of *C. scandens*, the original IR genes of the nine lineages had higher synonymous substitution rates ranging from 1.2 times to 24.5 times greater than those of their CRCIRs (**Figure S4**).

**Figure 3:**
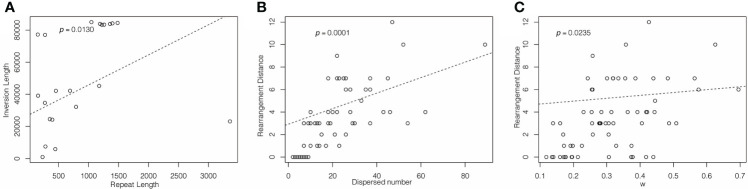
PGLS analysis of **(A)** dispersed repeat length and inversion length; **(B)** dispersed repeat number and rearrangement distance; **(C)** rearrangement distance and ω.

For IR-lacking species, the *d_S_* IR/*d_S_* SC values of the same kind of genes were about 1 (**Figure S5**). But the *rpl2* of two species, the *rpl23* of six species, the *ndhB* of two species, the *rps7* of one species, the *rps12* of two species, the *ycf1* of four species, and *ycf2* of three species had *d_S_* IR/*d_S_* SC values less than 0.5 (**Table S2**). And the *rpl2* of four species, the *rpl23* of three species, the *ndhB* of eight species, the *rps7* of six species, and the *rps12* of seven species had *d_S_* IR/*d_S_* SC values greater than 3.7 (**Table S2**).

### Changes of selection direction and intensity

The analysis using different branching models showed that the IR-lacking lineages were under purifying selection as a whole, and the selection was relaxed significantly compared with the background branches, except for Arecaceae (**Table 2**). However, when we classified protein-coding genes, we found that some kinds of genes had different selection direction and intensity (show detail in **Table S3**). More specially, we found that *rpl*, *rpo* and *rps* genes were under significantly relaxed purifying selection; only *rpl* and *rps* genes in Cactaceae were detected to be under significant positive selection (**Table S3**).

**Table 2:** Selectional strength and direction. ω_0_ indicates the result of the one-ratio model (all branches in the phylogenetic tree of this model are equal); ω_1_ indicates the result of the two-ratio model.

Lineage	ω0	ω1	*P-*value		Selection	Change
Arecaceae	0.4106	0.3777	0.6097	not significant	Negative	intensification
Cactaceae	0.1520	0.7628	0.0000	significant	Negative	relaxation
Geraniaceae	0.1799	0.2870	0.0000	significant	Negative	relaxation
Leguminosae	0.1612	0.2858	0.0000	significant	Negative	relaxation
*Camoensia*	0.2028	0.3950	0.0000	significant	Negative	relaxation
the putranjivoid clade	0.2042	0.4302	0.0000	significant	Negative	relaxation
*Passiflora*	0.1998	0.3226	0.0000	significant	Negative	relaxation
Cupressophyta	0.1905	0.3499	0.0000	significant	Negative	relaxation
Pinaceae	0.2157	0.4162	0.0000	significant	Negative	relaxation

*P-*value indicates the significance of LRTs of the one-ratio model against the two-ratio model.

### Statistics of indels and SNVs frequency

The indels frequency of plastomes of IR-lacking species was significantly higher than those of their CRCIRs (**Figures 1D** and **S6**). Specifically, the indels frequency in protein-coding genes, rRNA genes, and intergenic regions in IR-lacking species was 0.8–10.5, 1.0–15.9, and 0.8–2.2 times of those in CRCIRs (**Table S2**). Comparing with those of other IR-lacking species, the indels frequency of *T. spectabilis* was the lowest.

The SNVs frequency of most IR-lacking species was also higher than those of their CRCIRs (**Figures 1E**, **S6**). SNVs frequency was the lowest in rRNA genes and the highest in intergenic regions in both IR-lacking species and CRCIRs. The SNVs frequency of protein-coding genes, rRNA genes, and intergenic regions in IR-lacking species was 0.8–3.7, 0.9–13.1, and 0.8–3.1 times of those in CRCIRs (**Table S2**).

### GC content and codon usage

The GC content of IR-lacking species was significantly lower than those of their CRCIRs (**Figures 1F**, **S6**). Specifically, the GC content of IR-lacking species was in the range of 33.9%–39.5%, compared with a range of 35.8%–40.1% for their CRCIRs (**Table S2**). The GC content of protein-coding genes was also significantly lower than that of their CRCIRs (**Figures 1G**, **S6**). However, the GC3 content of IR-lacking species was significantly higher than that of their CRCIRs (**Figures 1H**, **S6**), the GC3 content of IR-lacking species was in the range of 23.9%–40.70%, compared with 24.7%–33.9% in their CRCIRs (**Table S2**)

There was no difference in the total number of codons used by IR-lacking species compared with their CRCIRs, nor was there any difference in the preferred codon usage per amino acid, but most of the IR-lacking species had slightly higher RSCU values (**Table S4**). Most of the preferred codon usage ended with A/T; only three codons end with G/C. However, *T. spectabilis* had five codons end with G/C (**Table S4**). Of the stop codons, TAA was more frequent than any other codon (**Table S4**).

### Phylogenetic ANOVA in Geraniaceae

**Figure 4:**
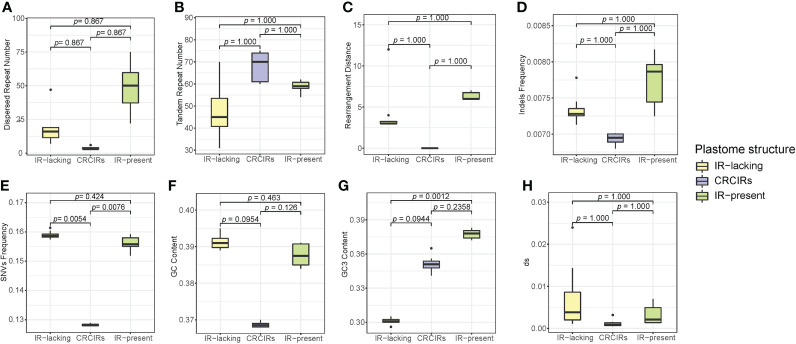
Boxplots for phylogenetic ANOVA statistical with post-hoc tests of eight variables between IR-lacking plastomes IR-present plastomes with variation and their CRCIRs. **(A)** dispersed repeat number; **(B)** tandem repeat number; **(C)** rearrangement distance; **(D)** indels frequency; **(E)** SNVs frequency; **(F)** whole plastome GC content; **(G)** GC3 content; **(H)** synonymous substitution rates.

Only SNVs frequency and GC3 content of ANOVA analyses actually showed a significant difference (**Figure 4**). For SNVs frequency, IR-lacking plastomes and CRCIRs plastomes (pairwise corrected *P-*values= 0.0054), and CRCIRs plastomes and IR-present plastomes (pairwise corrected *P-*value = 0.0076) showed significant differences; For GC3 content, IR-present plastomes and IR-lacking plastomes (pairwise corrected *P-*value = 0.0012) showed significant differences. However, the phylogenetic ANOVA performed on the other six variables (repeat number, rearrangement distance, indels frequency, GC content and *d_S_*) showed no significant difference among groups (**Figure 4**).

The differences of each variable of IR-lacking plastomes averaged 4.3 higher rearrangement distance, 19.6 less tandem repeats,14.6 more dispersed repeats, 0.0004 higher indels frequency, 0.03069 higher SNVs frequency, 0.00597 higher *d_S_*, 0.023 higher GC content and 0.05 lower GC3 content than those of CRCIRs plastomes; The differences of each variable of IR-lacking plastomes averaged 2.1 less rearrangement distance, 10.3 less tandem repeats, 30.6 less dispersed repeats, 0.0004 lower indels frequency, 0.0029 higher SNVs frequency, 0.00391 higher *d_S_*, 0.004 higher GC content and 0.076 lower GC3 content than those of IR-present plastomes; The differences of each variable of IR-present plastomes averaged 6.3 higher rearrangement distance, 9.3 less tandem repeats, 30 more dispersed repeats, 0.0008 higher indels frequency, 0.0278 higher SNVs frequency, 0.0045 higher *d_S_*, 0.01904 higher GC content and 0.026 higher GC3 content than those of CRCIRs plastomes (**Table S5**).

## Discussion

### Plastomes characterization of IR-lacking species

Among nine IR-lacking lineages, five lineages have lost IR_A_ and four lineages have lost IR_B_ (**Figure S1J**). The loss of one copy of IR appears to be a random event in a phylogenetic context. Variation in IR regions and gene loss are major contributors to the variation in plastome size and structure (Chen et al., 2021). Due to the loss of IR and some genes, the plastome length of the IR-lacking species is generally much shorter. Only one exception was detected, the IR-lacking plastome of *Agathis dammara* (145,625 bp) is larger than the plastomes of its four CRCIRs (136,689 bp–139,924 bp) because it has double *rRNA5* genes and more tandem repeats. Plastomes of the studied IR-lacking species, except for *T. spectabilis* (Arecaceae), have differing degrees of gene loss or pseudogenization. Although there were no genes consistently lost across all nine lineages, we found that the genes with the highest loss frequency are *rpl* and *rps* genes, which participate in translation and protein-modifying as large and small subunits of ribosomal protein (Bock, 2007; Wicke et al., 2011). The IR-lacking plastomes have also experienced frequent loss of *infA* and *accD*. The function of *infA* is as a translation initiation factor (Wicke et al., 2011). The function of *accD*, involving lipid acid synthesis, is also unrelated to photosynthesis (Wicke et al., 2011). Although IR-lacking species lose many genes, the loss of these genes does not destroy the core function of plastids. Therefore, IR-lacking species can function as fully autotrophic plants. In addition, plastids, as semi-autonomous organelles (Herber, 1962), are regulated by nuclear genes, and the function of these lost genes may be compensated by nuclear genes. In Cactaceae and Pinaceae, a large number of lost *ndh* genes are involved in the redox of photosynthetic NADH dehydrogenase (Bock, 2007), which may slightly fine-tune photosynthesis (Li et al., 2021). The reason for the loss of the *ndh* gene in these cases is still not yet clear. Some *ndh* genes may not be essential for plant survival, or their lost functions can be compensated by other factors (Suorsa et al., 2012; Strand et al., 2019). Except for *T. spectabilis*, all other IR-lacking species have lost the *clpP* intron 1 or both intron 1 and 2. We believe that this shared loss is probably biologically significant, but its cause and mechanism remain unexplained (Jansen et al., 2008; Wang et al., 2018; Bedoya et al., 2019; Jin et al., 2020a).

Many gene duplication events, frequently accompanying dispersed repeats, occurred in IR-lacking species (**Table S1**). *trnI-GAU* and *trnA-UGC*, located in the IR region, have the highest duplication frequency. Experiments involving diverse plants plastomes (Hotto et al., 2011; Zhelyazkova et al., 2012; Guo et al., 2015; Castandet et al., 2016) have shown that *trnI-GAU* and *trnA-UGC* are the most highly expressed genes (Mower and Vickrey, 2018). This increase in expression is likely driven by the presence of two gene copies (one in each IR region); this suggests that one of the main functions of IR may be to increase gene dosage (Mower and Vickrey, 2018). We also found the duplication of *rrn5* in *Agathis dammara* and *Sciadopitys verticillata*. Choi et al. (2019) also found two copies of three rRNA genes of *rrn4.5*, *rrn5* and *rrn23* in the plastomes of some *Medicago* spp. from the IRLC. Duplications of these original IR genes in the IR-lacking plastome may help to maintain gene expression levels.

### Significant structural variation in IR-lacking plastomes

Multiple studies have found increased structural variation in plastomes of IR-lacking lineages (Palmer and Thompson, 1981; Palmer and Thompson, 1982; Palmer et al., 1987; Guisinger et al., 2011; Wu et al., 2011a; Wu et al., 2011b; Choi et al., 2019; Jin et al., 2020a). This has led to the suggestion that IRs function to stabilize the structure of the plastome, perhaps by imposing structural constraints on the plastome, thereby impeding rearrangement events (Mower and Vickrey, 2018). Our study shows that all IR-lacking lineages have increased rearrangement compared with their CRCIRs, mainly attributable to inversions, ranging from 603 bp in *Agathis dammara* bp to 84,957 bp in *Drypetes indica*. Most plastomes of IR-lacking lineages have a significantly increased number of novel small, dispersed repeats (> 30 bp) compared with those of their CRCIRs. Many previous studies (e.g., Wu et al., 2011b; Qu et al., 2017; Ruhlman et al., 2017; Mower and Vickrey, 2018; Jin et al., 2020a; Lee et al., 2021) suggested the presence of smaller repeats is a major driver of plastomic rearrangements and the accumulation of novel small dispersed repeats may substitute for the function of original IR. Our study also detected a significant positive correlation between numbers of such dispersed repeats and the rearrangement distances (**Figure 3B**), and the rearrangement endpoints of approximately 15% of the inversions are associated with small, dispersed repeats. This suggests that repeat-mediated intra- and intermolecular recombination plays a major role in controlling plastome rearrangement. Moreover, we confirmed that longer dispersed repeats have a stronger ability to mediate inversion (**Figure 3A**).

A previous study speculated that increased genomic rearrangements could be explained by relaxed selection on variation caused by improper DNA repair (Guisinger et al., 2011). In this study, we confirmed that there was an obvious correlation between the rearrangement distance and overall selection pressure (**Figure 3C**). Although the selection pressure on different classes of genes is varied (**Table S3**), the selection pressure in the IR-lacking plastomes tends to be relaxed compared with that in plastomes of their CRCIRs (**Table 2**). This means the weaker the purifying selection pressure (more relaxed) on the plastid genome, the more drastic the change in plastome structure. This result is also consistent with the high frequency of indels and SNVs (**Figures 1D, E**), which may be due to the mutations introduced into plastomes (Kimura and Ota, 1971).

### Increased plastome sequence evolution in IR-lacking plastomes

Our study found that the genome-wide synonymous substitution rate of most IR-lacking species was accelerated (**Figure 2**). Change in synonymous substitution rate is often assumed to be neutral and dependent on base mutations (Kimura, 1983). Therefore, improper DNA repair also might be the reason for its acceleration (Guisinger et al., 2011). In addition, there was an order of magnitude difference in synonymous substitution rate between different lineages, which may be due to their different habit (herbs have higher *d_S_*) and age (Smith and Donoghue, 2008). The results of classification and calculation of protein-coding genes in SC region showed that the synonymous substitution rate of housekeeping genes accelerated higher than photosynthesis genes in IR-lacking species compared with their CRCIRs (**Table S2**). This is not surprising given that the main function of the chloroplast is photosynthesis (Wicke et al., 2011), which should result in more conservative evolution in photosynthesis genes.

Previous studies have shown that rates of nucleotide substitution are 3.7 times lower in IR genes compared with SC genes (Mower and Vickrey, 2018), which is generally considered to be a consequence of enhanced copy-correction activity in the IR (Wolfe et al., 1987; Perry and Wolfe, 2002; Zhu et al., 2016). This means that decreased substitution rates should follow gene transfer from the SC into the IR (Perry and Wolfe, 2002; Zhu et al., 2016) and increased substitution rates should follow the gene transfer from the IR into the SC (Li et al., 2016; Zhu et al., 2016). Consistent with previous studies, we found that the substitution rate significantly increased in the remaining copy of IRs in IR-lacking plastomes (e.g., Perry and Wolfe, 2002; Guisinger et al., 2008; Wu and Chaw, 2014; Qu et al., 2017), and about 74% of them achieve substitution rate levels of SC genes (**Figure S4**; **Figure S5** and **Table S2**). Previously, this acceleration has been considered as a direct result of IR copy loss following the loss of homologous recombination between two IR copies, rather than as an intrinsic property of these genes (Perry and Wolfe, 2002). Our results show the synonymous substitution rate of SC genes of IR-lacking plastomes and their CRCIRs plastomes are not significant different (**Figure S3** and **Table S2**).

Including the GC content (33.9%–39.5%) of our studied IR-lacking plastomes, the GC content is highly conserved in the plastomes of land plants, typically between 30–40% (Bock, 2007), which is affected by the error-checking bias of the DNA polymerase and/or efficiency for DNA denaturation during replication or transcription (Guisinger et al., 2011). The decrease in GC content may be detrimental to the stability of the sequence (Yakovchuk et al., 2006). The increase of GC3 content in IR-lacking plastomes confirm the “constraint model” proposed by Birdsell who thought the increase is due to the high recombination rate caused by the process of biased DNA repair (Genereux, 2002).

### Does IR promote plastome structure and sequence evolution?

The function of the IR remains elusive. All reported autotrophic species containing IR-lacking plastomes have normal phenotypes. The absence of an IR also does not necessarily impact a clade’s adaptability or speciation rate, as evidenced by the IRLC of Fabaceae, which contains ca. 5,400 species (International Legume Database and Information Service) spanning diverse habitat types around the globe.

Lee et al. (2021) questioned the function of the IR in maintaining plastome structure and even the function of IR per se. Our thorough analyses on IR-lacking autotrophic seed plant lineages and their CRCIRs found significant plastome structural rearrangement, gene loss/pseudogenization and duplication, accumulation of novel small repeats, and accelerated rates of substitution rates in IR-lacking lineages. However, five IR-lacking species have conservative structures (Palmer et al., 1987; Lee et al., 2021 and our results), and some IR-present lineages that have experienced IR-contraction or IR-expansion also have highly rearranged plastomes and increased rates of molecular evolution (Lin et al., 2012; Blazier et al., 2016; Weng et al., 2016). We found most variables of IR-lacking plastomes from *Erodium* and IR-present plastomes from *Geranium* were numerically larger than those of their CRCIRs, although the tandem repeat number of CRCIRs was higher than those of IR-lacking plastomes and IR-present plastomes, and GC3 content of CRCIRs was higher than those of IR-lacking plastomes but lower than those IR-present plastomes. However, our phylogenetic ANOVA results only showed significant differences among IR-lacking plastomes, IR-present plastomes and CRCIRs in three variables: SNVs, GC content, and GC3 content, and of those only SNVs were at greater frequency in both IR-lacking and IR-present plastomes compared to CRCIRs by *post-hoc* pairwise t-tests. The ANOVA results for the other variables might not have shown significant differences among groups due to limited sample sizes. Additionally, the IR-present plastomes had numerically higher rearrangement distance, dispersed repeat number, tandem repeat number, indels frequency and GC3 content than those of IR-lacking plastomes (**Figure 3**). In light of these results showing as much or greater structural and sequence instability in *Geranium* plastomes with the IR as in *Erodium* plastomes without it, we inferred IR loss may not be a direct driver of increased structural variation and sequence evolution in the IR-lacking plastomes, but that IR loss itself might be a result of other processes that generally impact structural variation and evolutionary rates across the entire plastome. Previous studies identified nuclear-encoded plastome DNA repair genes like CHM/MSH1 and RecA could suppress recombination between repeats, mutations in these genes could increase plastome rearrangements and nucleotide substitutions (Guisinger et al., 2011). Loss of function in nuclear-encoded DNA repair genes may cause more structural rearrangements and increased molecular evolution in IR-lacking plastomes, and the loss of the IR may thus be an accompanying event involving the process of nucleocytoplasmic co-evolution.

## Data availability statement

The data presented in this study are deposited in the GenBank repository, accession number ON009072-ON009081 and ON022041.

## Author contributions

T-SY and Z-XW designed the study. Z-XW and D-JW contributed to experiments, sequences, and data analysis. Z-XW, D-JW, and T-SY wrote and edited the manuscript; all authors commented on the manuscript. All authors contributed to the article and approved the submitted version.

## Funding

This project was funded by grants from the National Natural Science Foundation of China key international (regional) cooperative research project (No. 31720103903); the Science and Technology Basic Resources Investigation Program of China (2019FY100900), the Strategic Priority Research Program of the Chinese Academy of Sciences (XDB31010000); the Large-scale Scientific Facilities of the Chinese Academy of Sciences (No. 2017-LSF-GBOWS-02); the National Natural Science Foundation of China (Project No. 31270274); the Yunling International High-end Experts Program of Yunnan Province, China (YNQR-GDWG-2017-002 and YNQR-GDWG-2018-012).

## Acknowledgments

We thank the Kunming Institute of Botany, Royal Botanic Gardens Kew, Xishuangbanna Tropical Botanical Garden, Australian National Botanic Gardens, Jilin University and Royal Botanic Gardens Edinburgh for providing samples; the Molecular Biology Experiment Center, Germplasm Bank of Wild Species in Southwest China for laboratory assistance; and the iFlora High Performance Computing Center of Germplasm Bank of Wild Species (iFlora HPC Center of GBOWS, KIB, CAS) for data processing. Also, we thank Gregory Wakely Stull for his detail revisions on our manuscript; Joseph L.M. Charboneau from the University of Arizona for his help on data analyses; Ming Yan from Nanjing Forestry University, An-Dan Zhu, Wen-Shu Fan, Chao-Nan Fu, Qin Tian, Yunxia Li, Lu Gan, Rong Zhang, Shui-Yin Liu from Kunming Institute of Botany for their suggestions on this study.

## Conflict of interest

The authors declare that the research was conducted in the absence of any commercial or financial relationships that could be construed as a potential conflict of interest.

## Publisher’s note

All claims expressed in this article are solely those of the authors and do not necessarily represent those of their affiliated organizations, or those of the publisher, the editors and the reviewers. Any product that may be evaluated in this article, or claim that may be made by its manufacturer, is not guaranteed or endorsed by the publisher.

## Supplementary material

Supplementary Figure 1 The ML phylogenetic tree of **(A)** Cupressophyta; **(B)** Pinaceae; **(C)** IRLC; **(D)** the putranjivoid clade; **(E)** Cactaceae; **(F)** Geraniaceae; **(G)** *Passiflora*; **(H)** Arecaceae; **(I)** *Camoensia*; **(J)** all IR-lacking species and their CRCIRs; **(K)** 21 IR-lacking species listed in ; **(L)** 22 Geraniaceae species listed in **Table S5** based on protein-coding and rRNA genes matrix. The number at each node indicates the ML bootstrap values.

Supplementary Figure 2 Plastid genome variation in the nine lineages. **(A)** Arecaceae; **(B)** Cactaceae; **(C)** the putranjivoid clade; **(D)** *Passiflora*; **(E)** IRLC; **(F)** *Camoensia*; **(G)** Geraniaceae; **(H)** Cupressophyta; **(I)** Pinaceae. Whole-plastome alignments divide the plastid genome of our study taxa into different Locally Collinear Blocks (LCB), which are shown as color-coded representations of syntenic regions. The IR_A_ or IR_B_ was removed from plastid genomes with two copies of the large inverted repeats to allow for an optimal homology assessment. Blocks below the horizontal central line represent inversions relative to the references. The height of the colored region within a block reflects the average sequence identity relative to the reference. Species names are color-coded to indicate their family: references species (black), CRCIRs species (blue), and IR-lacking species (red). The pink blocks in both IR-present species indicate the IR regions. Red blocks represent *rrn5*, *rrn4.5*, *rrn23*, and *rrn16* genes, green blocks represent *trnA-UGC* and *trnI-GAU* genes.

Supplementary Figure 3 Boxplots for comparisons of synonymous substitution rates of SC region protein-coding genes between IR-lacking species and their CRCIRs with phylogenetic t-test. **(A)** Cupressophyta; **(B)** Pinaceae; **(C)** IRLC; **(D)** the putranjivoid clade; **(E)** Cactaceae; **(F)** Geraniaceae; **(G)** *Passiflora*; **(H)** Arecaceae; **(I)** *Camoensia.*

Supplementary Figure 4 Boxplots for comparisons of synonymous substitution rates of original IR protein-coding genes between IR-lacking species and their CRCIRs with phylogenetic t-test. **(A)** Cupressophyta; **(B)** Pinaceae; **(C)** IRLC; **(D)** the putranjivoid clade; **(E)** Cactaceae; **(F)** Geraniaceae; **(G)** *Passiflora*; **(H)** Arecaceae; **(I)** *Camoensia.*

Supplementary Figure 5 Frequency distribution histogram of the *d_S_* IR/*d_S_* SC values.

Supplementary Figure 6 Boxplots for comparisons of eight variables between IR-lacking species and their CRCIRs from each of nine lineages with phylogenetic t-test. (A) dispersed repeat number; (B) tandem repeat number; (C) rearrangement distance; (D) indels frequency; (E) SNVs frequency; (F) whole plastome GC content; (G) protein-coding genes GC content; (H) GC3 content.

Supplementary Table 1 Sampling information and plastome characteristics.

Supplementary Table 2 The raw data of rearrangement distance, terminal branch ω, indels frequency, SNVs frequency, repeats number and length, *d_S_*, whole plastome GC content, protein-coding genes GC content and GC3 content.

Supplementary Table 3 Selectional strength and direction of protein-coding genes.

Supplementary Table 4 Codon usage.

Supplementary Table 5 The data of rearrangement distance, indels frequency, SNVs frequency, repeats number, *d_S_*, whole plastome GC content and GC3 content for phylogenetic ANOVA analysis in Geraniaceae.

Supplementary Table 6 Specimens voucher and source information.
