# Supplementary material for: Does IR-loss promote plastome structural variation and sequence evolution?
Source: Front Plant Sci. 2022 Sep 29;13:888049. doi: 10.3389/fpls.2022.888049 (PMC9560873; doi:10.3389/fpls.2022.888049)
Supplement: Supplementary Figure 1 — The ML phylogenetic tree of (A) Cupressophyta; (B) Pinaceae; (C) IRLC; (D) the putranjivoid clade; (E) Cactaceae; (F) Geraniaceae; (G) Passiflora; (H) Arecaceae; (I) Camoensia; (J) all IR-lacking species and their CRCIRs; (K) 21 IR-lacking species listed in ; (L) 22 Geraniaceae species listed in Table S5 based on protein-coding and rRNA genes matrix. The number at each node indicates the ML bootstrap values. [file DataSheet_1.zip › supplementary figures/Figure S6.pdf]

(A)

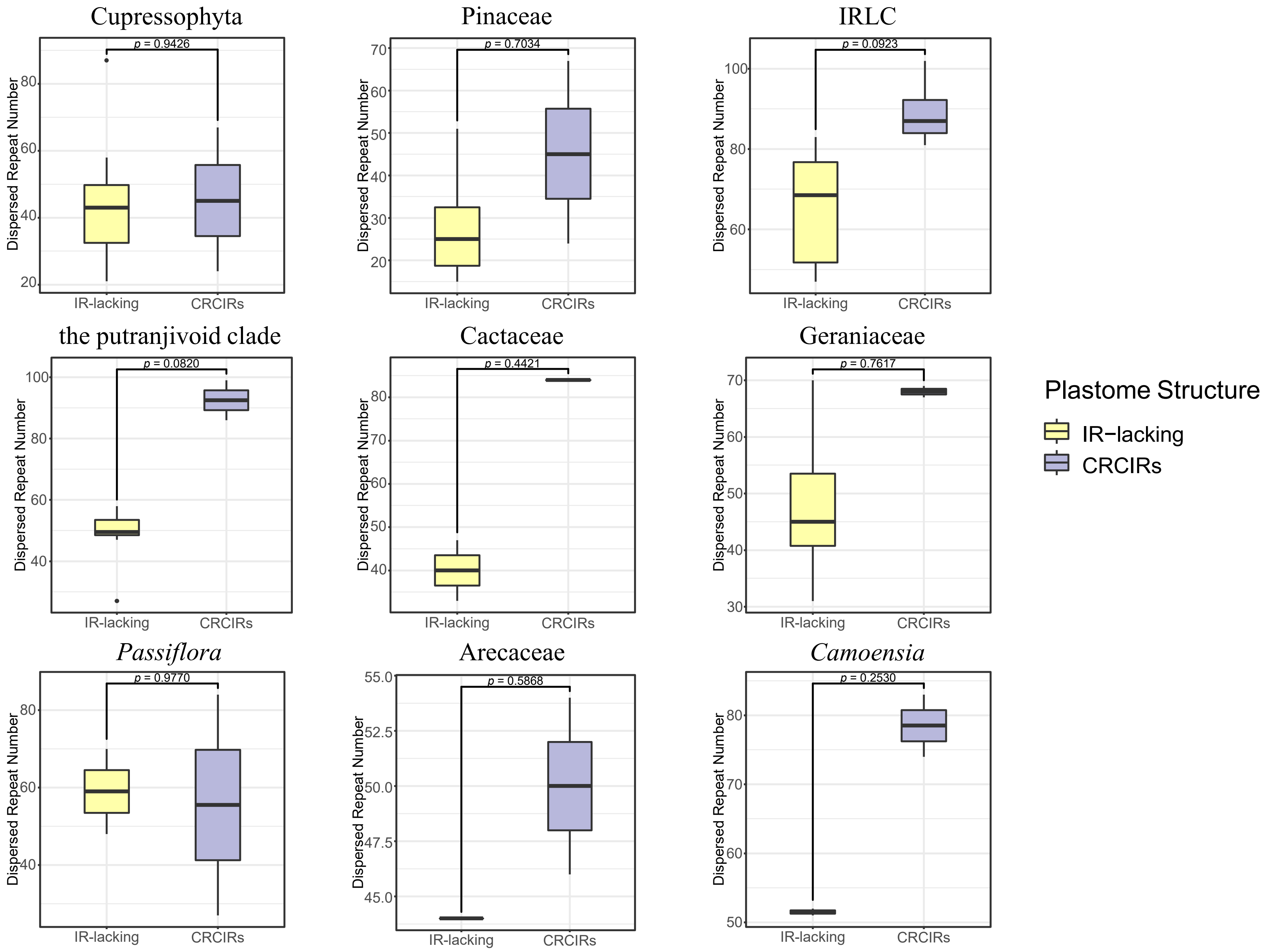
Cupressophyta
p = 0.9426
Pinaceae
p = 0.7034
IRLC
p = 0.0923
the putranjivoid clade
p = 0.0820
Cactaceae
p = 0.4421
Geraniaceae
p = 0.7617
Passiflora
p = 0.9770
Arecaceae
p = 0.5868
Camoensia
p = 0.2530
Dispersed Repeat Number
IR-lacking
CRCIRs
Plastome Structure
IR−lacking
CRCIRs

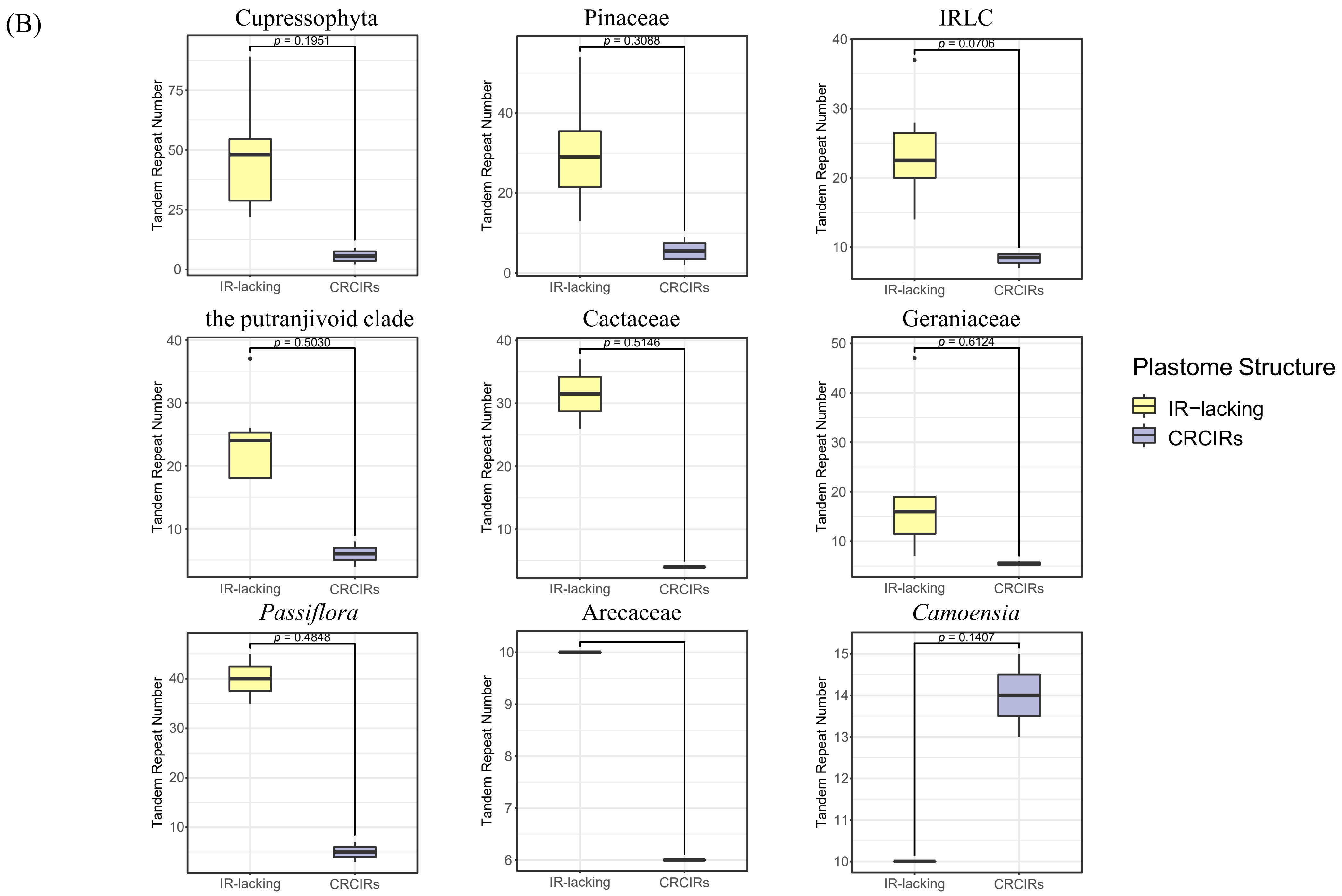
(B)
Cupressophyta
p = 0.1951
Pinaceae
p = 0.3088
IRLC
p = 0.0706
the putranjivoid clade
p = 0.5030
Cactaceae
p = 0.5146
Geraniaceae
p = 0.6124
Passiflora
p = 0.4848
Arecaceae
Camoensia
p = 0.1407
Tandem Repeat Number
IR-lacking
CRCIRs
Plastome Structure
IR−lacking
CRCIRs

(C)

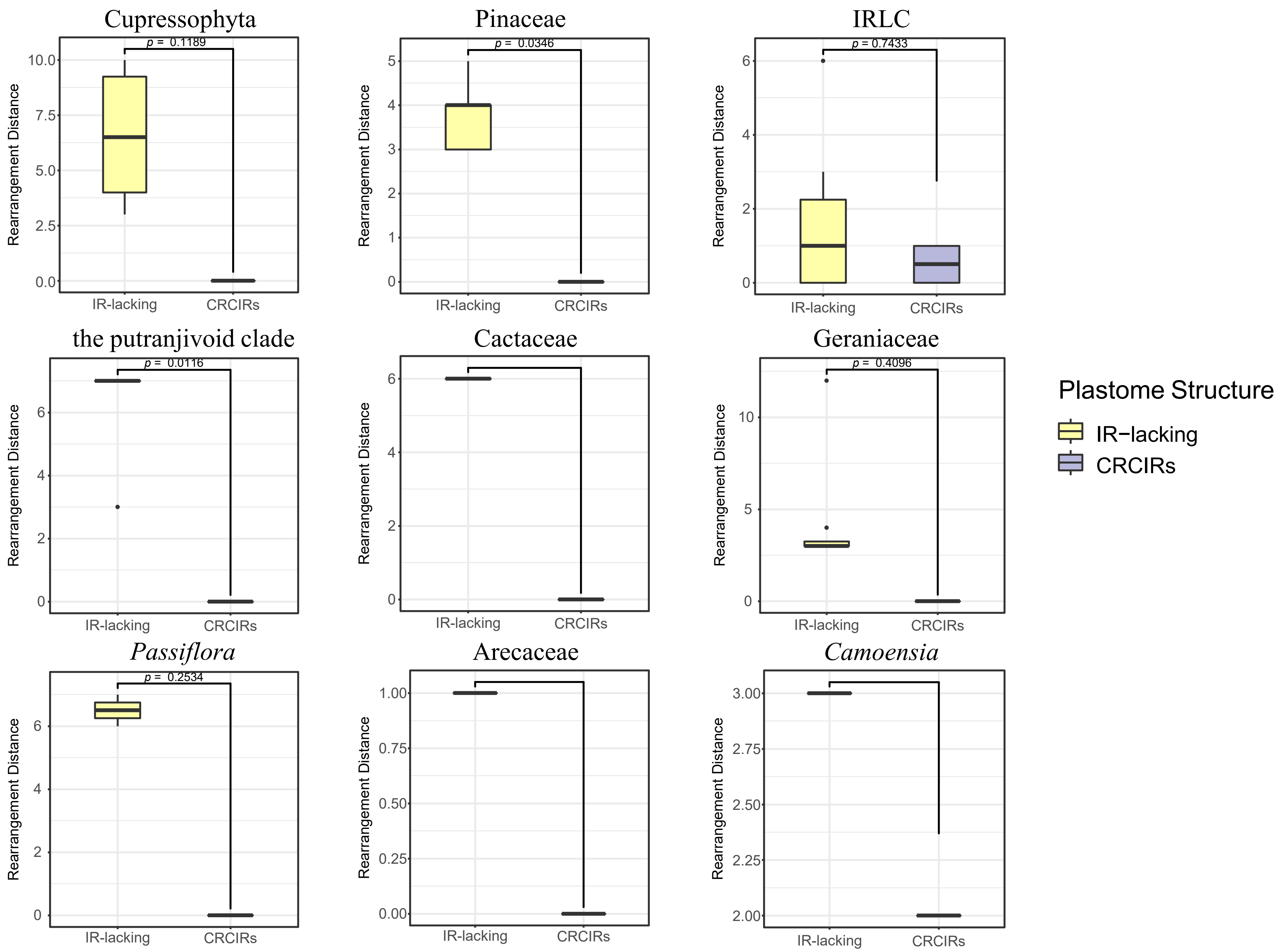
Cupressophyta
p = 0.1189
Pinaceae
p = 0.0346
IRLC
p = 0.7433
the putranjivoid clade
p = 0.0116
Cactaceae
Geraniaceae
p = 0.4096
Passiflora
p = 0.2534
Arecaceae
Camoensia
Rearrangement Distance
IR-lacking
CRCIRs
Plastome Structure
IR−lacking
CRCIRs

(D)

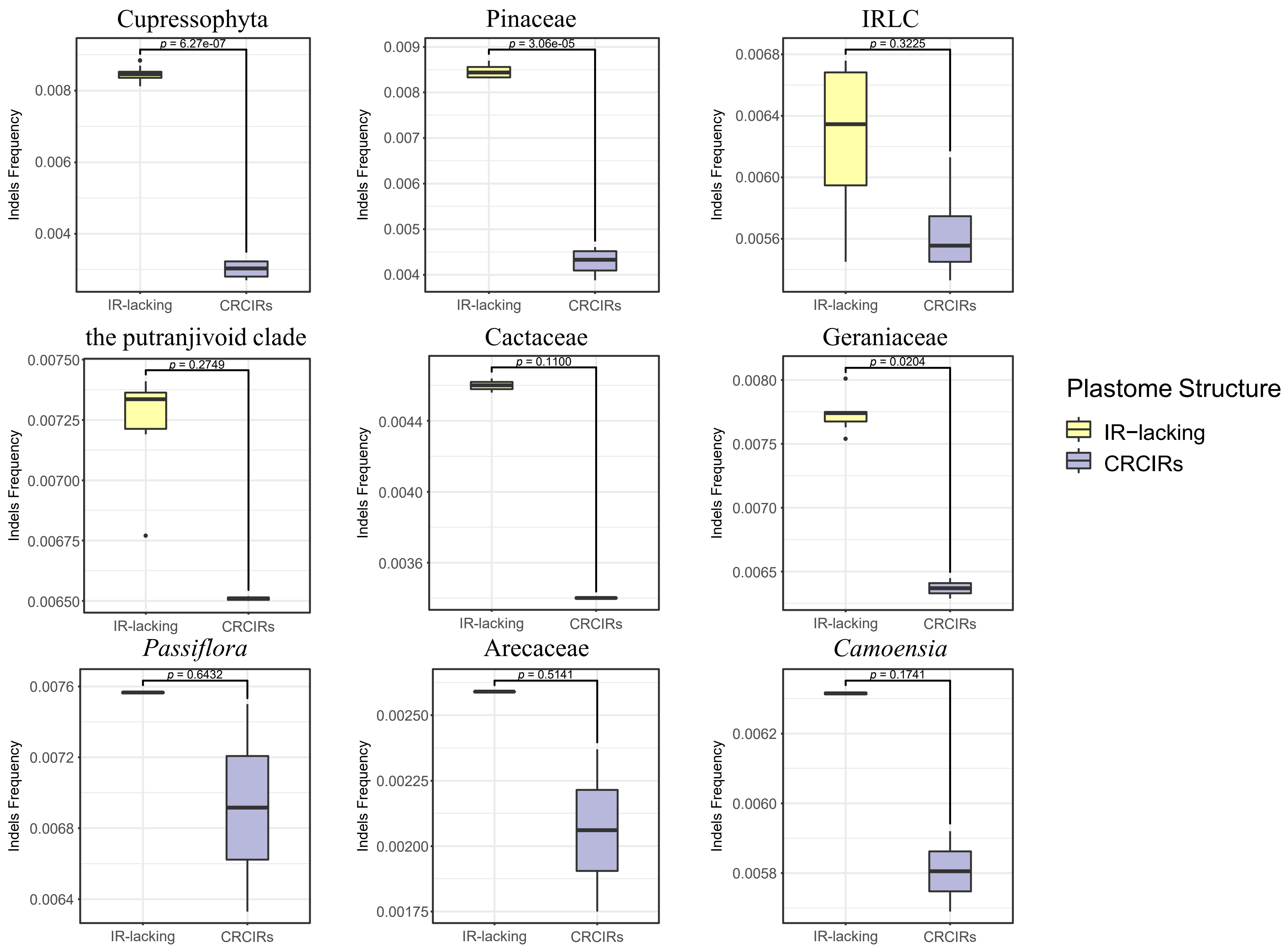
Cupressophyta
p = 6.27e-07
Pinaceae
p = 3.06e-05
IRLC
p = 0.3225
the putranjivoid clade
p = 0.2749
Cactaceae
p = 0.1100
Geraniaceae
p = 0.0204
Passiflora
p = 0.6432
Arecaceae
p = 0.5141
Camoensia
p = 0.1741
Indels Frequency
IR-lacking
CRCIRs
Plastome Structure
IR−lacking
CRCIRs

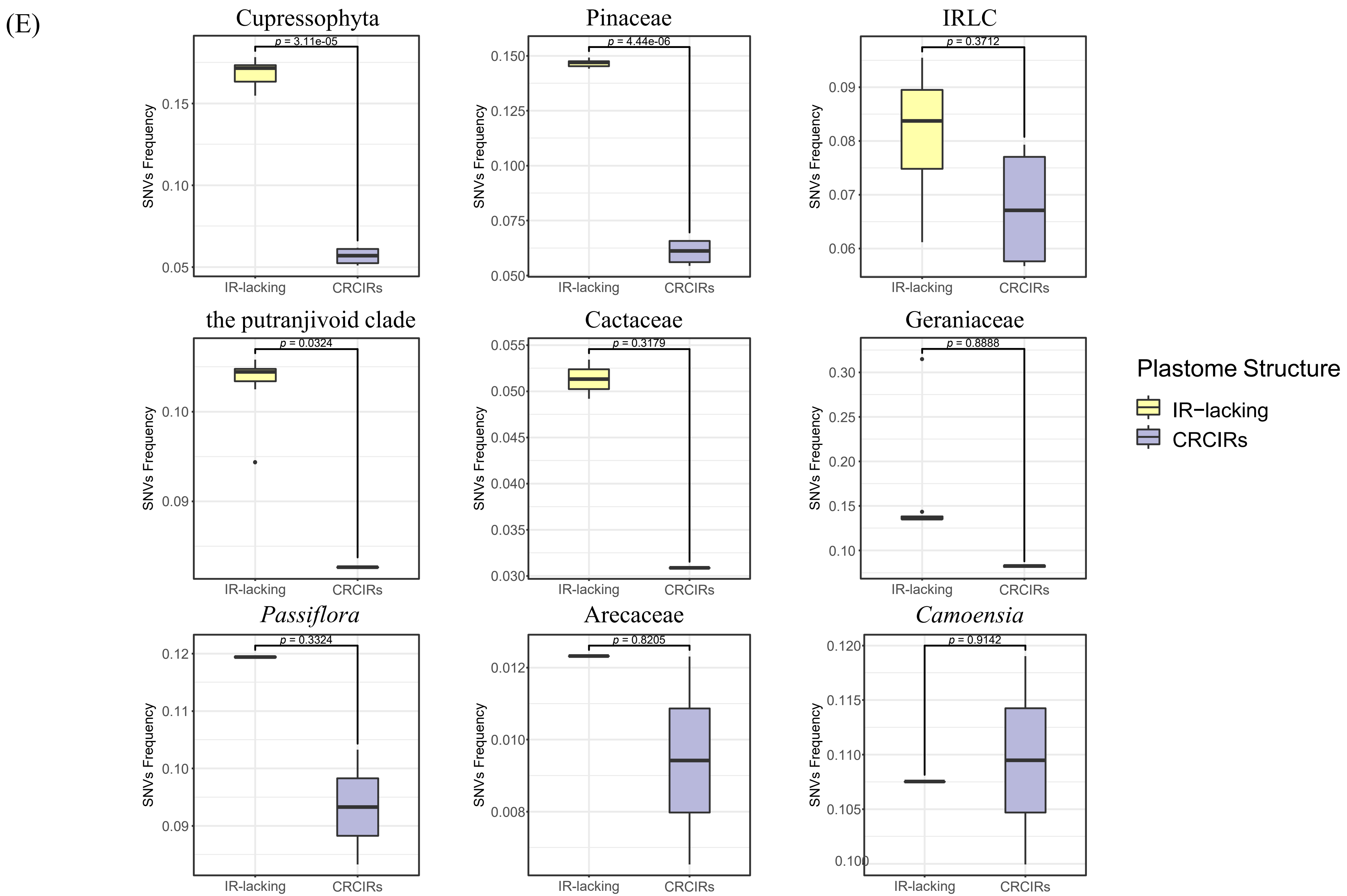
(E)
Cupressophyta
p = 3.11e-05
Pinaceae
p = 4.44e-06
IRLC
p = 0.3712
the putranjivoid clade
p = 0.0324
Cactaceae
p = 0.3179
Geraniaceae
p = 0.8888
Passiflora
p = 0.3324
Arecaceae
p = 0.8205
Camoensia
p = 0.9142
SNVs Frequency
IR-lacking
CRCIRs
Plastome Structure
IR−lacking
CRCIRs

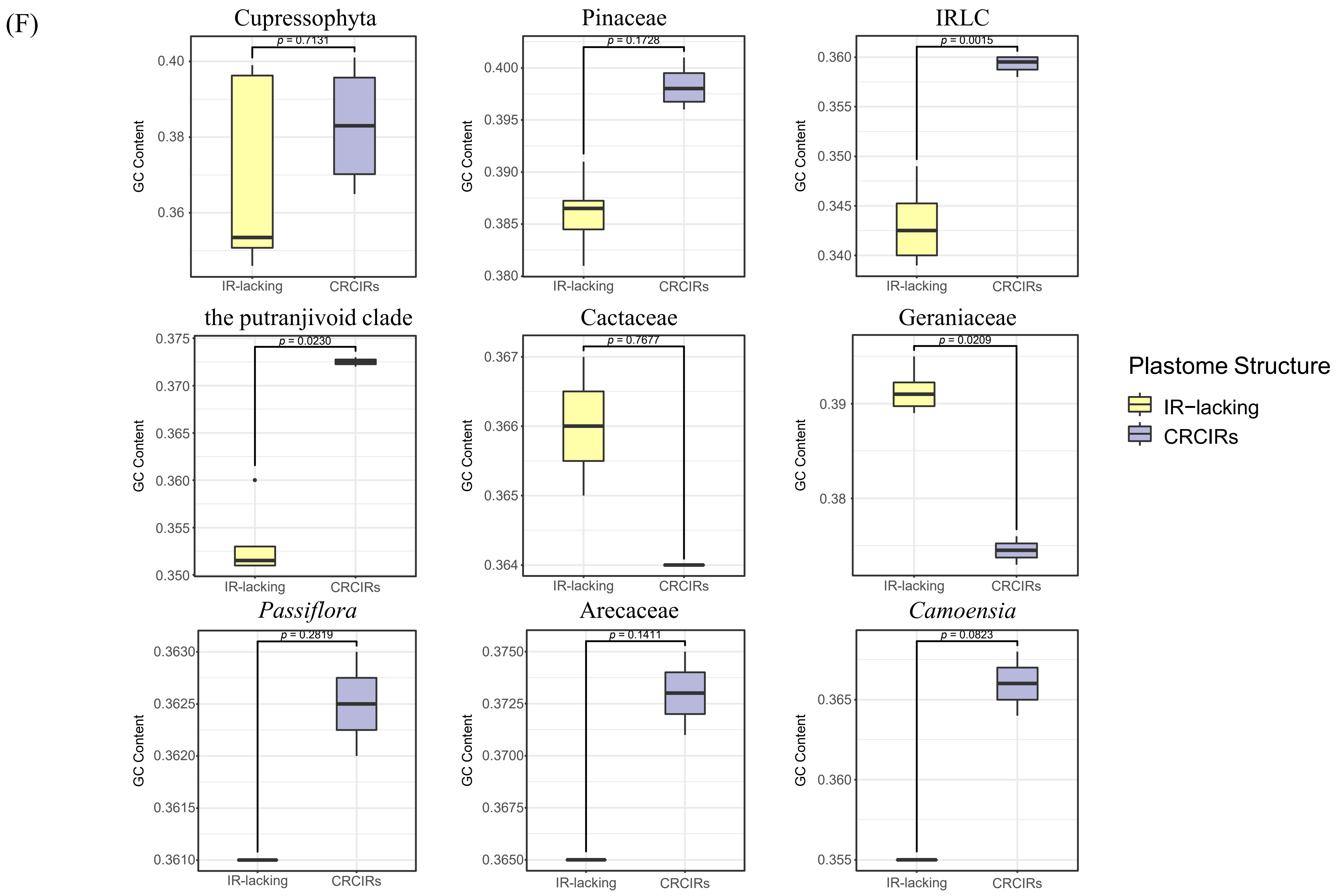

(F)
Cupressophyta
p = 0.7131
Pinaceae
p = 0.1728
IRLC
p = 0.0015
the putranjivoid clade
p = 0.0230
Cactaceae
p = 0.7677
Geraniaceae
p = 0.0209
Passiflora
p = 0.2819
Arecaceae
p = 0.1411
Camoensia
p = 0.0823
GC Content
IR-lacking
CRCIRs
Plastome Structure
IR−lacking
CRCIRs

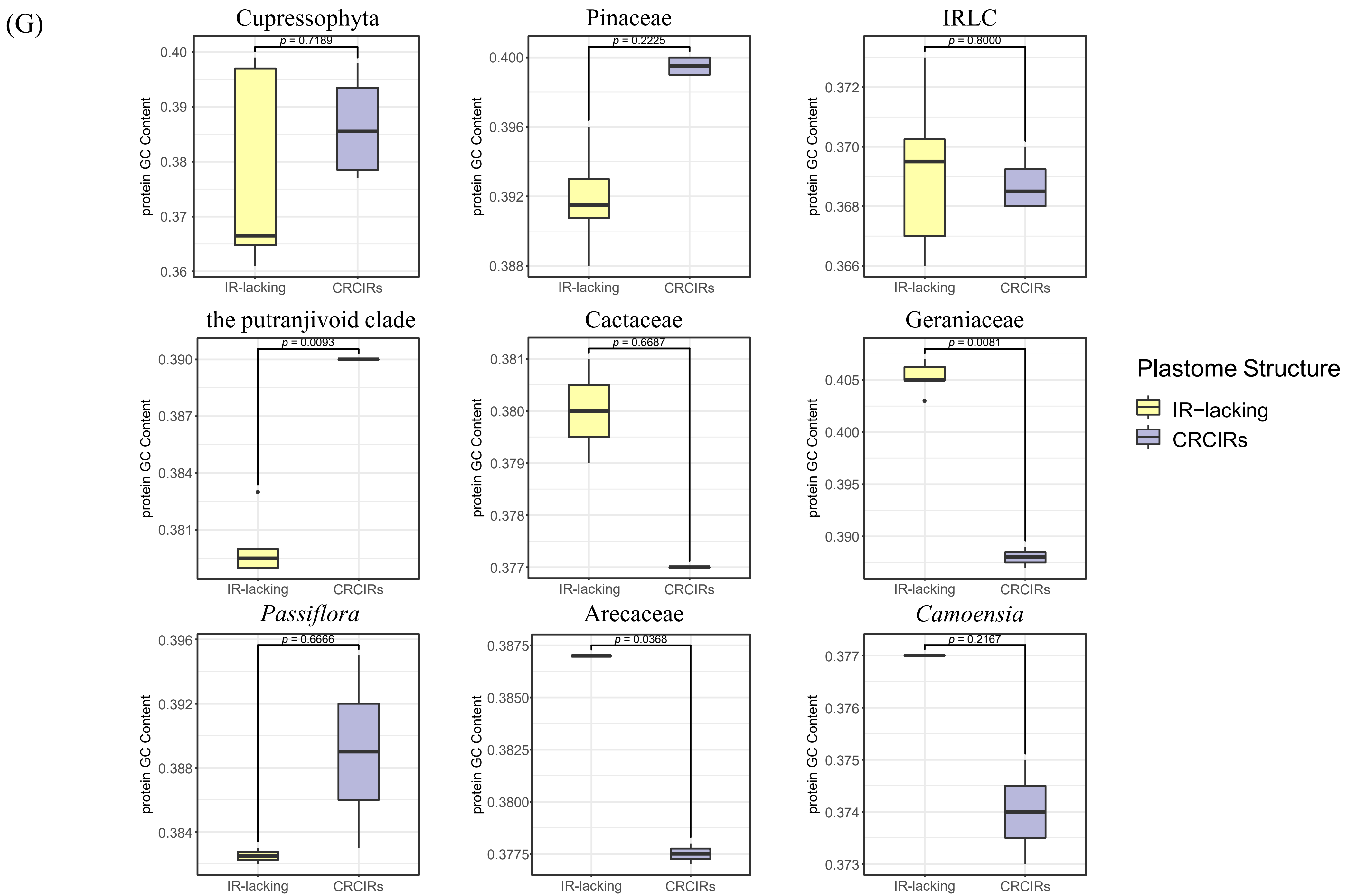
(G)
Cupressophyta
p = 0.7189
Pinaceae
p = 0.2225
IRLC
p = 0.8000
the putranjivoid clade
p = 0.0093
Cactaceae
p = 0.6687
Geraniaceae
p = 0.0081
Passiflora
p = 0.6666
Arecaceae
p = 0.0368
Camoensia
p = 0.2167
protein GC Content
IR-lacking
CRCIRs
Plastome Structure
IR−lacking
CRCIRs

(H)

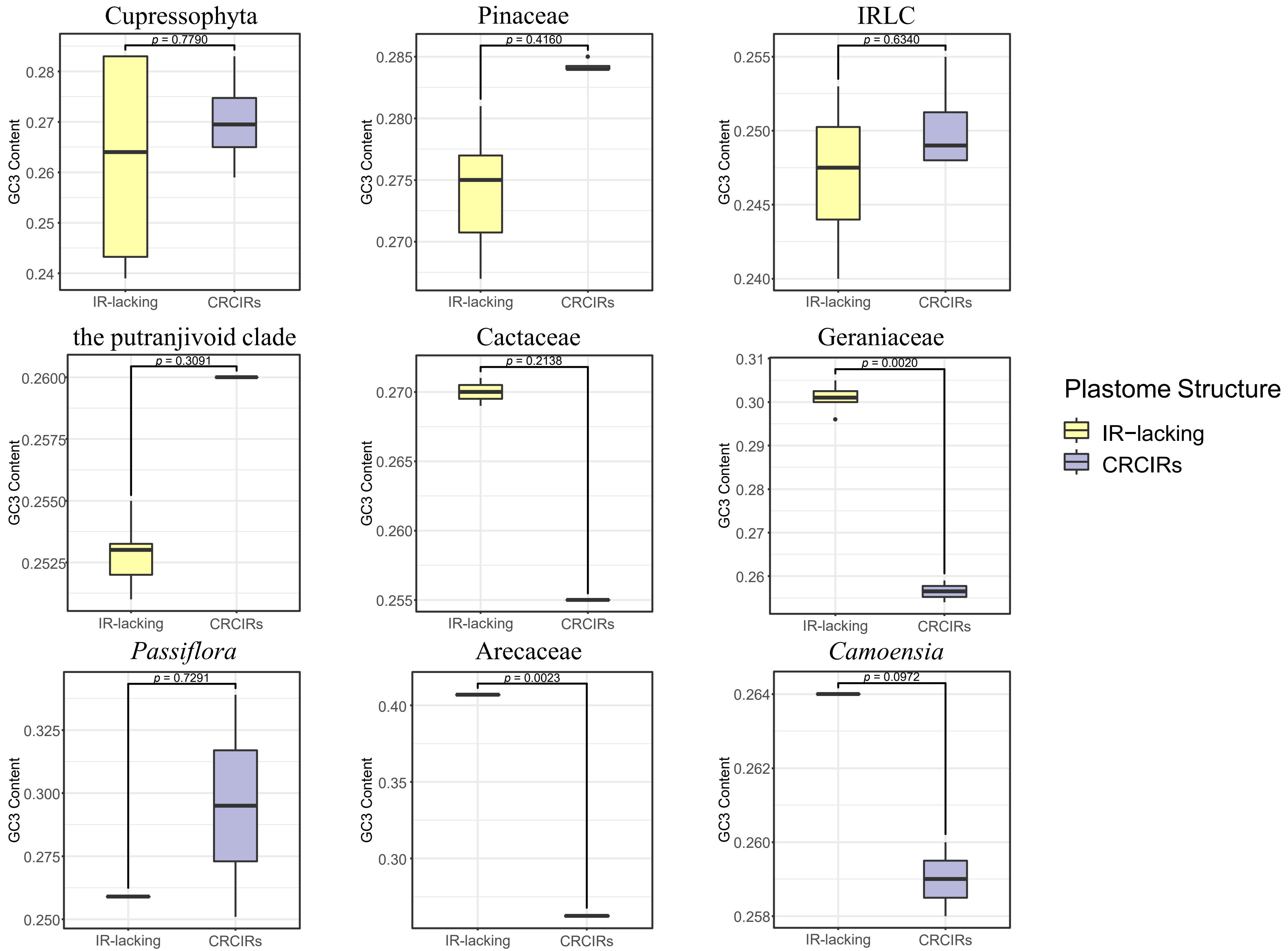
Cupressophyta
p = 0.7790
Pinaceae
p = 0.4160
IRLC
p = 0.6340
the putranjivoid clade
p = 0.3091
Cactaceae
p = 0.2138
Geraniaceae
p = 0.0020
Passiflora
p = 0.7291
Arecaceae
p = 0.0023
Camoensia
p = 0.0972
GC3 Content
IR-lacking
CRCIRs
Plastome Structure
IR−lacking
CRCIRs
