# Supplementary material for: Does IR-loss promote plastome structural variation and sequence evolution?
Source: Front Plant Sci. 2022 Sep 29;13:888049. doi: 10.3389/fpls.2022.888049 (PMC9560873; doi:10.3389/fpls.2022.888049)
Supplement: Supplementary Figure 1 — The ML phylogenetic tree of (A) Cupressophyta; (B) Pinaceae; (C) IRLC; (D) the putranjivoid clade; (E) Cactaceae; (F) Geraniaceae; (G) Passiflora; (H) Arecaceae; (I) Camoensia; (J) all IR-lacking species and their CRCIRs; (K) 21 IR-lacking species listed in ; (L) 22 Geraniaceae species listed in Table S5 based on protein-coding and rRNA genes matrix. The number at each node indicates the ML bootstrap values. [file DataSheet_1.zip › supplementary figures/Figure S4.pdf]

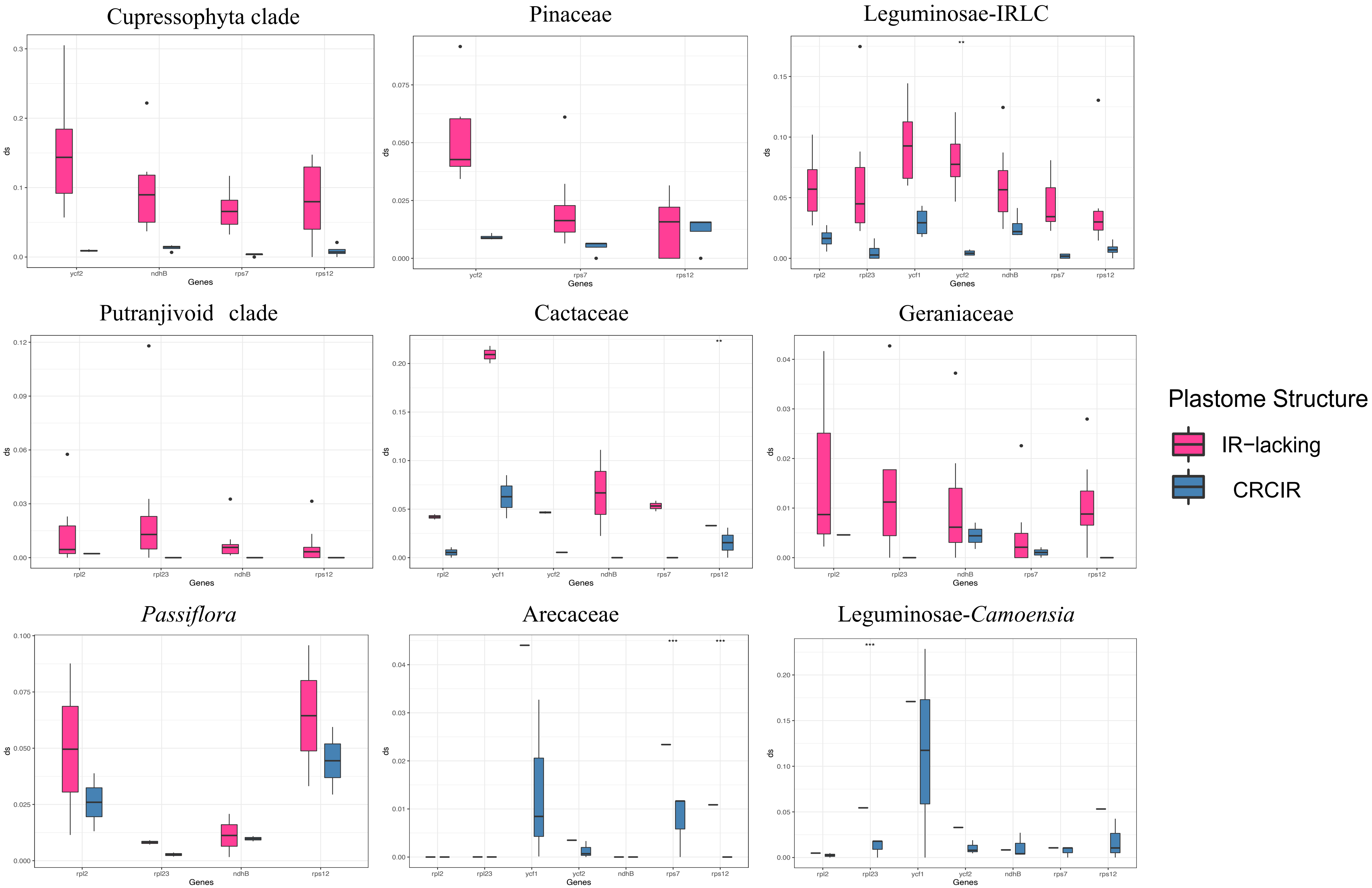
Cupressophyta clade
Pinaceae
Leguminosae-IRLC
Putranjivoid clade
Cactaceae
Geraniaceae
Passiflora
Arecaceae
Leguminosae-Camoensia
ds
Genes
ycf2
ndhB
rps7
rps12
rpl2
rpl23
ycf1
**
***
Plastome Structure
IR−lacking
CRCIR
