# Supplementary material for: Does IR-loss promote plastome structural variation and sequence evolution?
Source: Front Plant Sci. 2022 Sep 29;13:888049. doi: 10.3389/fpls.2022.888049 (PMC9560873; doi:10.3389/fpls.2022.888049)
Supplement: Supplementary Figure 1 — The ML phylogenetic tree of (A) Cupressophyta; (B) Pinaceae; (C) IRLC; (D) the putranjivoid clade; (E) Cactaceae; (F) Geraniaceae; (G) Passiflora; (H) Arecaceae; (I) Camoensia; (J) all IR-lacking species and their CRCIRs; (K) 21 IR-lacking species listed in ; (L) 22 Geraniaceae species listed in Table S5 based on protein-coding and rRNA genes matrix. The number at each node indicates the ML bootstrap values. [file DataSheet_1.zip › supplementary figures/Figure S1.zip!/Figure S1/J.pdf]

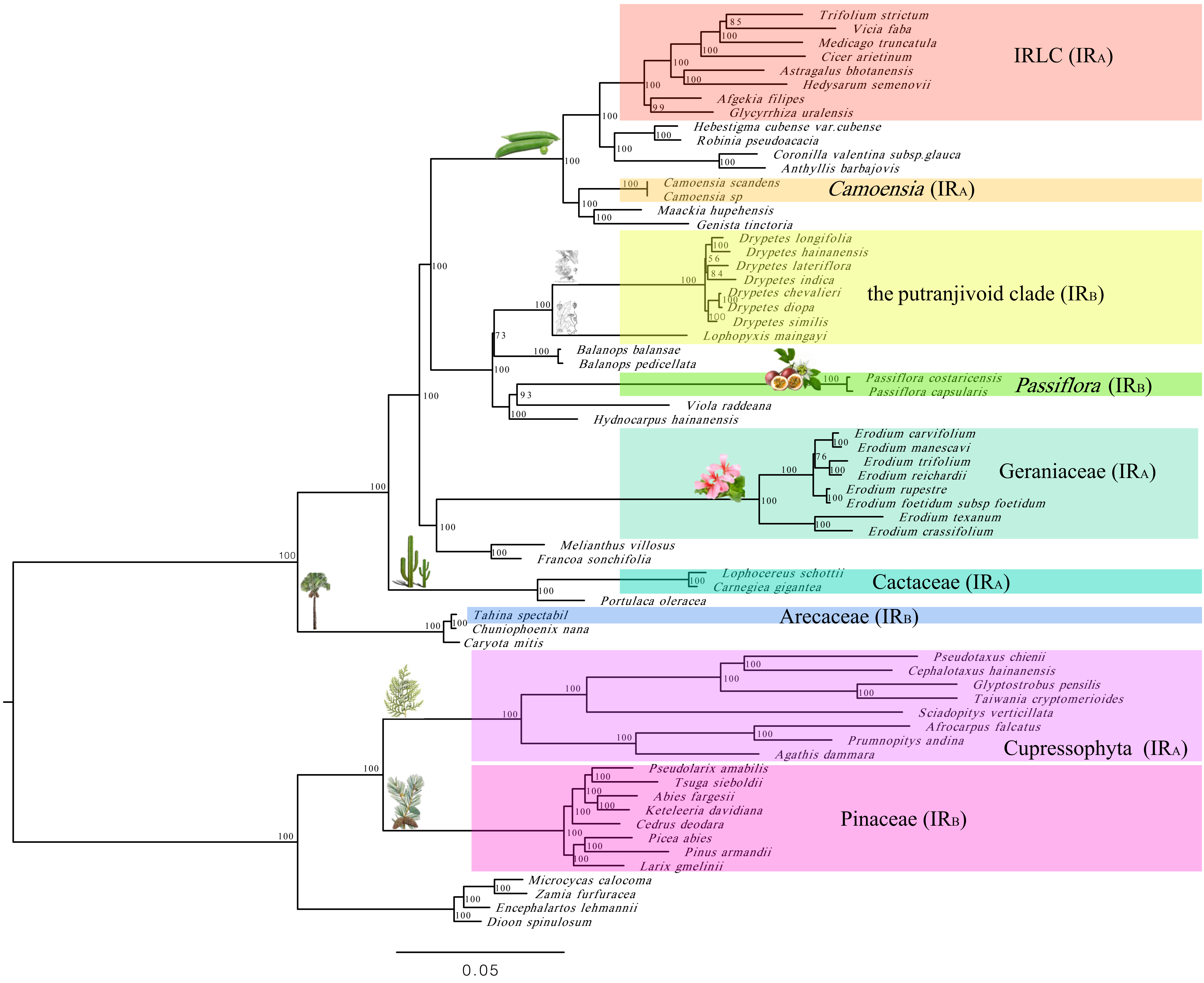
Trifolium strictum
Vicia faba
Medicago truncatula
Cicer arietinum
Astragalus bhotanensis
Hedysarum semenovii
Afgekia filipes
Glycyrrhiza uralensis
IRLC (IRA)
Hebestigma cubense var.cubense
Robinia pseudoacacia
Coronilla valentina subsp.glauca
Anthyllis barbajovis
Camoensia scandens
Camoensia sp
Camoensia (IRA)
Maackia hupehensis
Genista tinctoria
Drypetes longifolia
Drypetes hainanensis
Drypetes lateriflora
Drypetes indica
Drypetes chevalieri
Drypetes diopa
Drypetes similis
Lophopyxis maingayi
the putranjivoid clade (IRB)
Balanops balansae
Balanops pedicellata
Passiflora costaricensis
Passiflora capsularis
Passiflora (IRB)
Viola raddeana
Hydnocarpus hainanensis
Erodium carvifolium
Erodium manescavi
Erodium trifolium
Erodium reichardii
Erodium rupestre
Erodium foetidum subsp foetidum
Erodium texanum
Erodium crassifolium
Geraniaceae (IRA)
Melianthus villosus
Francoa sonchifolia
Lophocereus schottii
Carnegiea gigantea
Cactaceae (IRA)
Portulaca oleracea
Tahina spectabil
Chuniophoenix nana
Caryota mitis
Arecaceae (IRB)
Pseudotaxus chienii
Cephalotaxus hainanensis
Glyptostrobus pensilis
Taiwania cryptomerioides
Sciadopitys verticillata
Afrocarpus falcatus
Prumnopitys andina
Agathis dammara
Cupressophyta (IRA)
Pseudolarix amabilis
Tsuga sieboldii
Abies fargesii
Keteleeria davidiana
Cedrus deodara
Picea abies
Pinus armandii
Larix gmelinii
Pinaceae (IRB)
Microcycas calocoma
Zamia furfuracea
Encephalartos lehmannii
Dioon spinulosum
0.05
