# Supplementary material for: Does IR-loss promote plastome structural variation and sequence evolution?
Source: Front Plant Sci. 2022 Sep 29;13:888049. doi: 10.3389/fpls.2022.888049 (PMC9560873; doi:10.3389/fpls.2022.888049)
Supplement: Supplementary Figure 1 — The ML phylogenetic tree of (A) Cupressophyta; (B) Pinaceae; (C) IRLC; (D) the putranjivoid clade; (E) Cactaceae; (F) Geraniaceae; (G) Passiflora; (H) Arecaceae; (I) Camoensia; (J) all IR-lacking species and their CRCIRs; (K) 21 IR-lacking species listed in ; (L) 22 Geraniaceae species listed in Table S5 based on protein-coding and rRNA genes matrix. The number at each node indicates the ML bootstrap values. [file DataSheet_1.zip › supplementary figures/Figure S1.zip!/Figure S1/K.pdf]

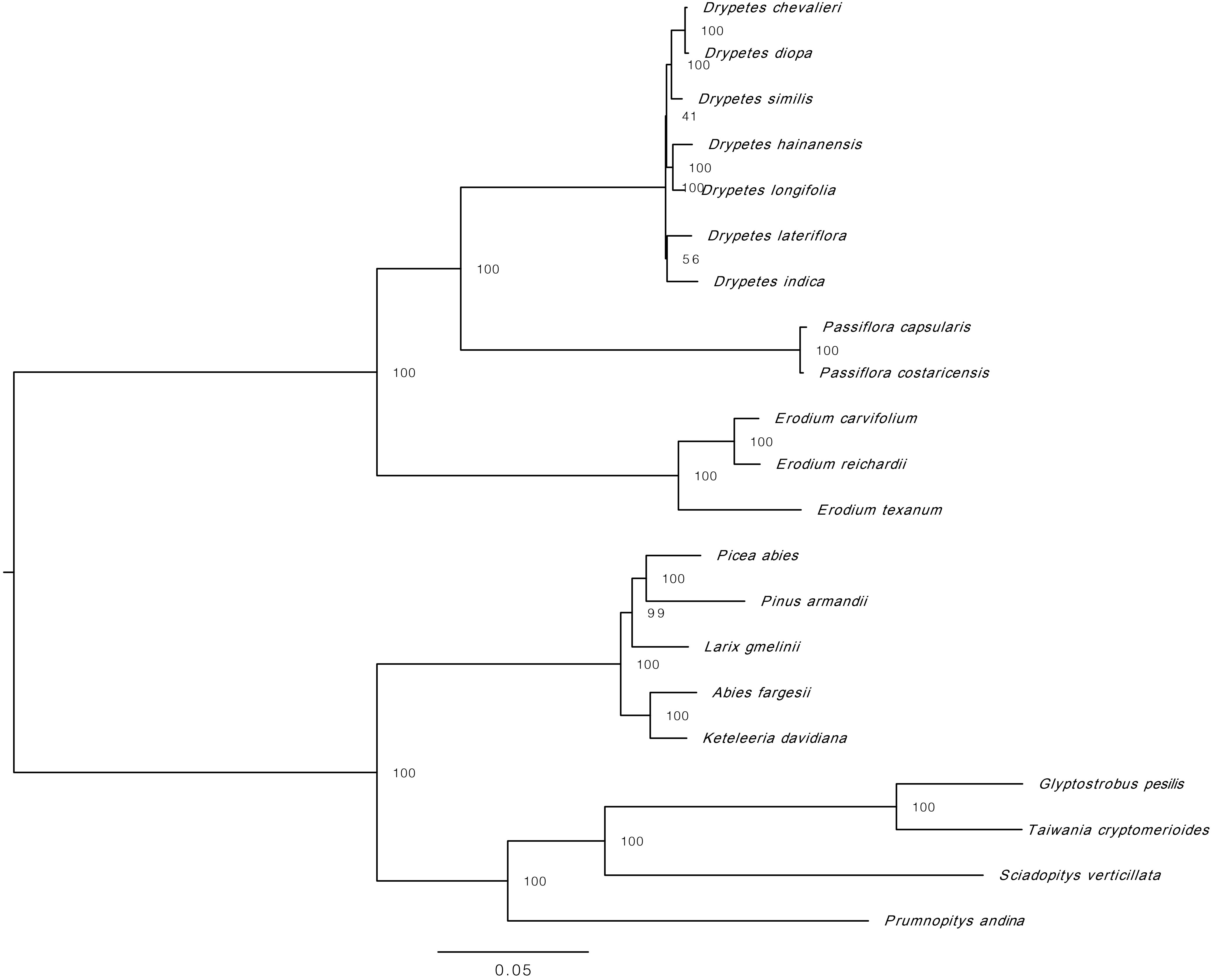

Drypetes chevalieri
100
Drypetes diopa
100
Drypetes similis
41
Drypetes hainanensis
100
100
Drypetes longifolia
Drypetes lateriflora
56
Drypetes indica
100
Passiflora capsularis
100
Passiflora costaricensis
100
Erodium carvifolium
100
Erodium reichardii
100
Erodium texanum
Picea abies
100
Pinus armandii
99
Larix gmelinii
100
Abies fargesii
100
Keteleeria davidiana
100
Glyptostrobus pesilis
100
Taiwania cryptomerioides
100
Sciadopitys verticillata
100
Prumnopitys andina
0.05
