# Supplementary material for: Does IR-loss promote plastome structural variation and sequence evolution?
Source: Front Plant Sci. 2022 Sep 29;13:888049. doi: 10.3389/fpls.2022.888049 (PMC9560873; doi:10.3389/fpls.2022.888049)
Supplement: Supplementary Figure 1 — The ML phylogenetic tree of (A) Cupressophyta; (B) Pinaceae; (C) IRLC; (D) the putranjivoid clade; (E) Cactaceae; (F) Geraniaceae; (G) Passiflora; (H) Arecaceae; (I) Camoensia; (J) all IR-lacking species and their CRCIRs; (K) 21 IR-lacking species listed in ; (L) 22 Geraniaceae species listed in Table S5 based on protein-coding and rRNA genes matrix. The number at each node indicates the ML bootstrap values. [file DataSheet_1.zip › supplementary figures/Figure S1.zip!/Figure S1/L.pdf]

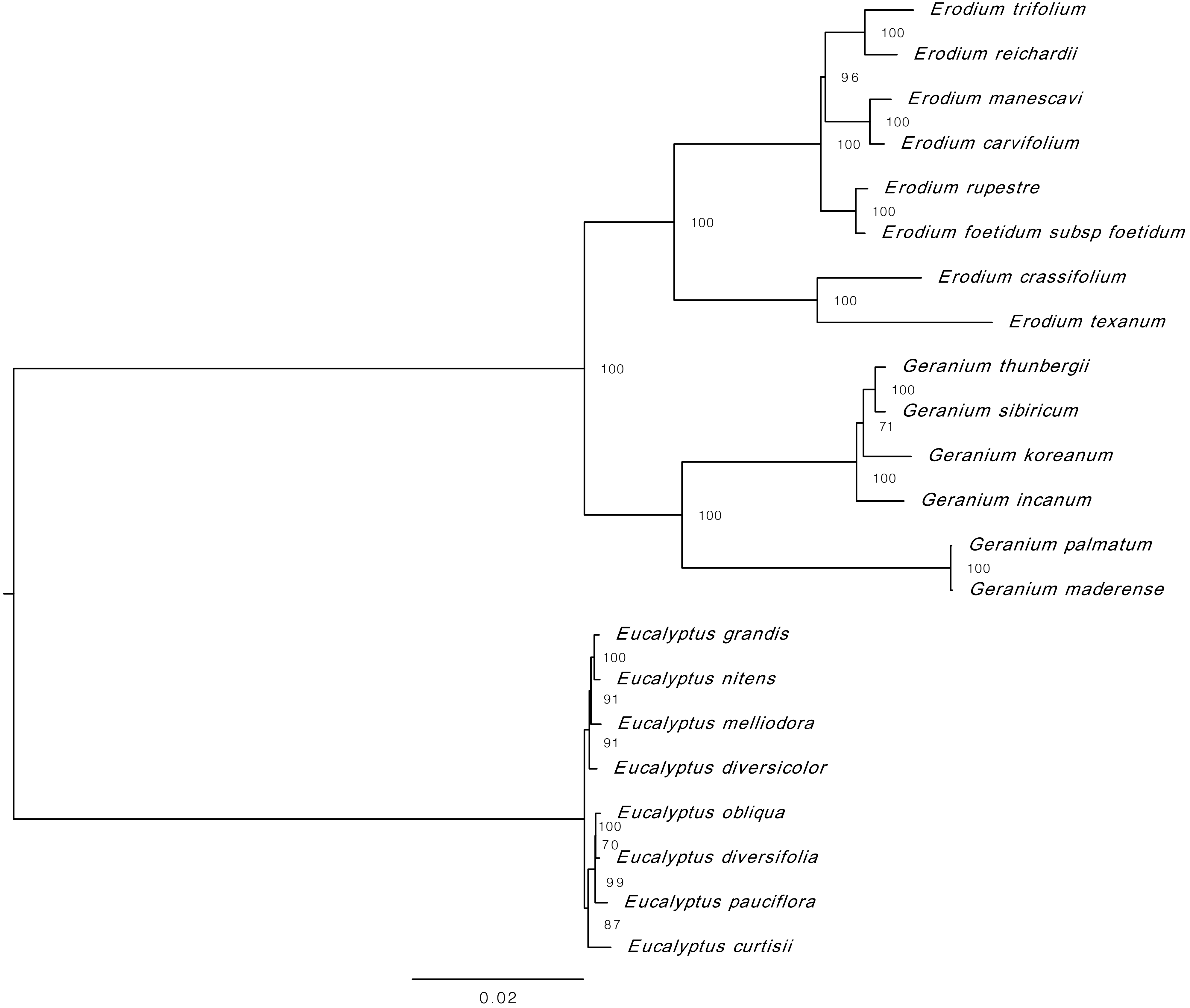
Erodium trifolium
100
Erodium reichardii
96
Erodium manescavi
100
Erodium carvifolium
100
Erodium rupestre
100
Erodium foetidum subsp foetidum
100
Erodium crassifolium
100
Erodium texanum
100
Geranium thunbergii
100
Geranium sibiricum
71
Geranium koreanum
100
Geranium incanum
100
Geranium palmatum
100
Geranium maderense
Eucalyptus grandis
100
Eucalyptus nitens
91
Eucalyptus melliodora
91
Eucalyptus diversicolor
Eucalyptus obliqua
100
70
Eucalyptus diversifolia
99
Eucalyptus pauciflora
87
Eucalyptus curtisii
0.02
