# Supplementary material for: Does IR-loss promote plastome structural variation and sequence evolution?
Source: Front Plant Sci. 2022 Sep 29;13:888049. doi: 10.3389/fpls.2022.888049 (PMC9560873; doi:10.3389/fpls.2022.888049)
Supplement: Supplementary Figure 1 — The ML phylogenetic tree of (A) Cupressophyta; (B) Pinaceae; (C) IRLC; (D) the putranjivoid clade; (E) Cactaceae; (F) Geraniaceae; (G) Passiflora; (H) Arecaceae; (I) Camoensia; (J) all IR-lacking species and their CRCIRs; (K) 21 IR-lacking species listed in ; (L) 22 Geraniaceae species listed in Table S5 based on protein-coding and rRNA genes matrix. The number at each node indicates the ML bootstrap values. [file DataSheet_1.zip › supplementary figures/Figure S1.zip!/Figure S1/A.pdf]

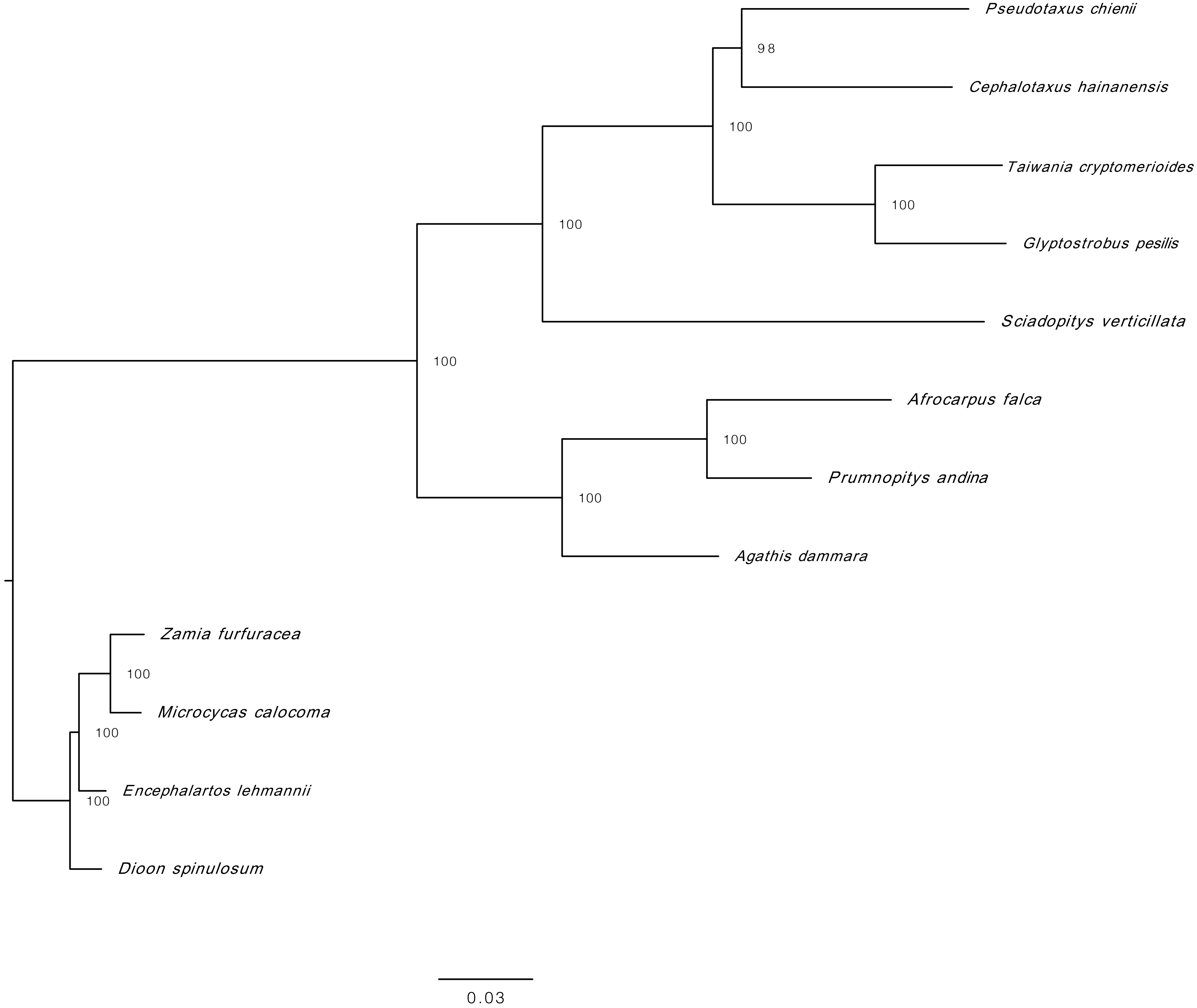
Pseudotaxus chienii
98
Cephalotaxus hainanensis
100
Taiwania cryptomerioides
100
Glyptostrobus pesilis
100
Sciadopitys verticillata
100
Afrocarpus falca
100
Prumnopitys andina
100
Agathis dammara
Zamia furfuracea
100
Microcycas calocoma
100
Encephalartos lehmannii
100
Dioon spinulosum
0.03
