# Supplementary material for: Does IR-loss promote plastome structural variation and sequence evolution?
Source: Front Plant Sci. 2022 Sep 29;13:888049. doi: 10.3389/fpls.2022.888049 (PMC9560873; doi:10.3389/fpls.2022.888049)
Supplement: Supplementary Figure 1 — The ML phylogenetic tree of (A) Cupressophyta; (B) Pinaceae; (C) IRLC; (D) the putranjivoid clade; (E) Cactaceae; (F) Geraniaceae; (G) Passiflora; (H) Arecaceae; (I) Camoensia; (J) all IR-lacking species and their CRCIRs; (K) 21 IR-lacking species listed in ; (L) 22 Geraniaceae species listed in Table S5 based on protein-coding and rRNA genes matrix. The number at each node indicates the ML bootstrap values. [file DataSheet_1.zip › supplementary figures/Figure S1.zip!/Figure S1/C.pdf]

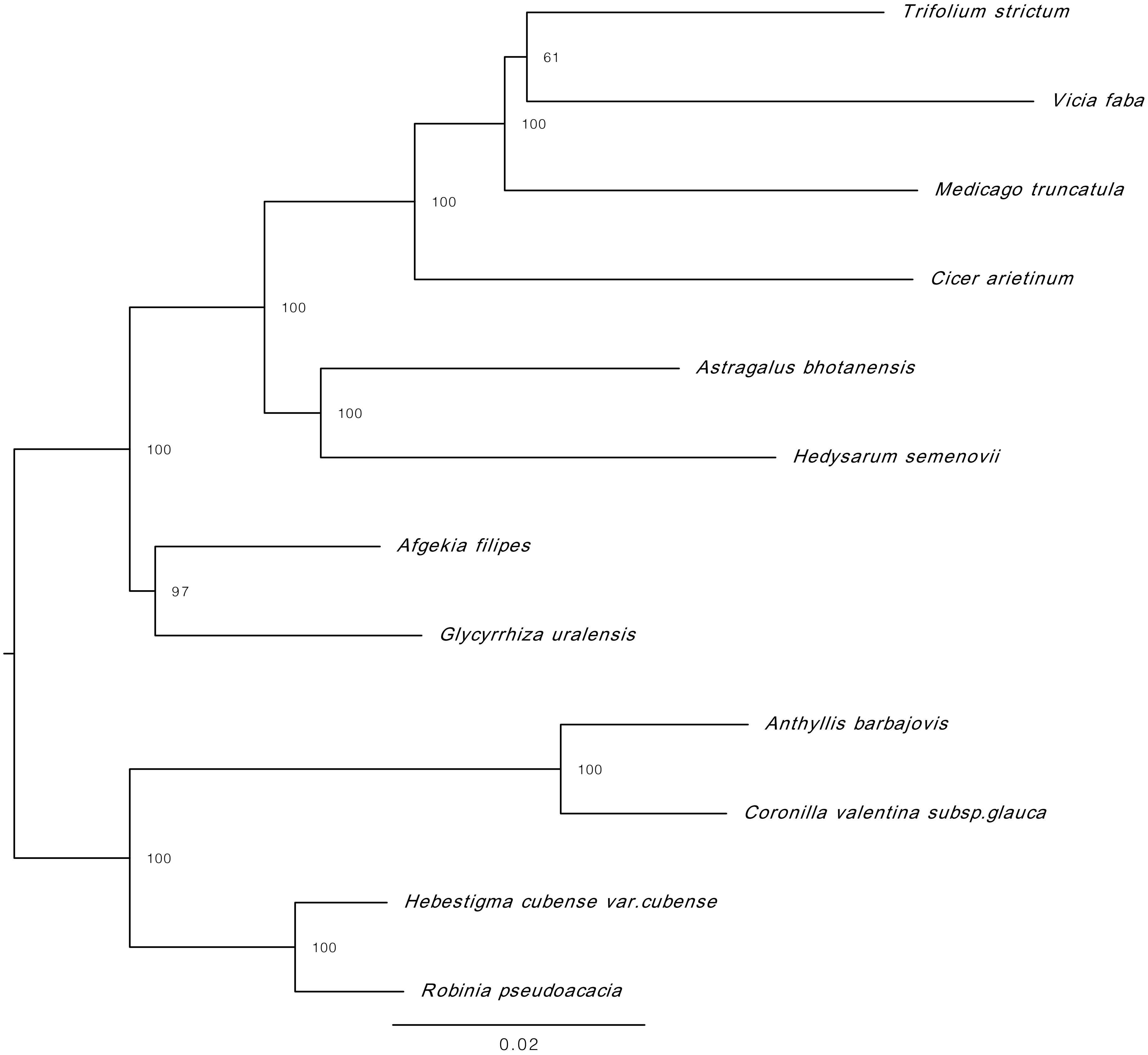
Trifolium strictum
61
Vicia faba
100
Medicago truncatula
100
Cicer arietinum
100
Astragalus bhotanensis
100
Hedysarum semenovii
100
Afgekia filipes
97
Glycyrrhiza uralensis
Anthyllis barbajovis
100
Coronilla valentina subsp.glauca
100
Hebestigma cubense var.cubense
100
Robinia pseudoacacia
0.02
